# Evolution and function of calponin and transgelin

**DOI:** 10.3389/fcell.2023.1206147

**Published:** 2023-06-08

**Authors:** Tzu-Bou Hsieh, J.-P. Jin

**Affiliations:** ^1^ Department of Obstetrics and Gynecology, Wayne State University School of Medicine, Detroit, MI, United States; ^2^ Department of Physiology and Biophysics, University of Illinois at Chicago College of Medicine, Chicago, IL, United States

**Keywords:** calponin, transgelin, isoform genes, evolution, structure-function relationship, cell motility, cytoskeleton

## Abstract

Calponin and transgelin (originally named SM22) are homologous cytoskeleton proteins that regulate actin-activated myosin motor functions in smooth muscle contraction and non-muscle cell motility during adhesion, migration, proliferation, phagocytosis, wound healing, and inflammatory responses. They are abundant cytoskeleton proteins present in multiple cell types whereas their physiological functions remain to be fully established. This focused review summarizes the evolution of genes encoding calponin and transgelin and their isoforms and discusses the structural similarity and divergence in vertebrate and invertebrate species in the context of functions in regulating cell motility. As the first literature review focusing on the evolution of the calponin-transgelin family of proteins in relevance to their structure-function relationship, the goal is to outline a foundation of current knowledge for continued investigations to understand the biological functions of calponin and transgelin in various cell types during physiological and pathological processes.

## 1 Introduction

Calponin and transgelin (originally named SM22) are homologous cytoskeleton proteins that regulate actin-activated myosin motor functions in smooth muscle contraction and non-muscle cell motility during adhesion, migration, proliferation, phagocytosis, wound healing, inflammatory response and fibrotic tissue remodeling (Liu and Jin, 2016a). Calponin and transgelin have each evolved into three homologous isoforms in vertebrates and their physiological functions remain to be fully established.

The primary structures of calponin isoforms have been determined in multiple vertebrate species. Their highly conserved core structure consists of a calponin homology (CH) domain, two actin-binding motifs and three calponin-like (CLIK) repeats. In comparison, the structure of transgelins consists of one CH domain, one actin-binding motif and one CLIK motif. The three vertebrate calponin isoforms are diverged in their C-terminal structures whereas transgelins lack the C-terminal variable region (Liu et al., 2017).

Homologs to vertebrate calponin and transgelin have been identified in invertebrate species. For example, a calponin-like protein was found in mussels as a regulatory protein for actin and tropomyosin-related cellular functions. It is larger than vertebrate calponins containing a CH domain and five CLIK repeats (Dobrzhanskaya et al., 2013; Sirenko et al., 2013). Several other calponin-like proteins have been reported in invertebrates containing a CH domain and various numbers of CLIK repeats ranging from 7 in tapeworm to 23 in mollusks (Ono, 2021). The calponin-like protein, unc-87, of *Caenorhabditis elegans* contains 7 CLIK repeats in the absence of the CH domain, possibly representing an ancestral form of calponin and transgelin family proteins (Yamashiro et al., 2007; Ono et al., 2015).

To understand the function of the conserved core structure of calponin-transgelin family proteins as well as the significantly diverged structures of their isoforms is of physiological and medical importance. To systemically review the molecular evolution of calponin and transgelin in vertebrate and invertebrate species can provide insights into the structure-function relationship of calponin and transgelin and the molecular mechanisms in their regulation of cell motility-related processes. Extracted from the current knowledge of calponin and transgelin structures in the sequence databases that have rapidly enriched in recent years, this focused review summarizes for the first time the molecular evolution of the calponin-transgelin gene family in the context of the structure-function relationships of calponin and transgelin and their isoforms across invertebrate and vertebrate species. The goal is to lay a foundation for better understanding the biological functions of calponin and transgelin in various cell types as a regulator of physiological and pathological processes.

## 2 Three calponin isoforms in vertebrates

Calponin was first identified in chicken gizzard smooth muscle in 1986 as abundant actin thin filament-binding protein with a potential function in the regulation of smooth muscle contraction (Takahashi et al., 1986; Takahashi et al., 1988). This smooth muscle calponin was later classified as calponin 1 after two other calponin isoforms were identified. Now we know that three homologous genes are present in vertebrates encoding three calponin isoforms (Jin et al., 2008). *CNN1* encodes calponin 1 (previously annotated as basic calponin based on its basic isoelectric point) (Gao et al., 1996). *CNN2* encodes calponin 2 (previously named neutral calponin) (Strasser et al., 1993; Masuda et al., 1996). *CNN3* encodes calponin 3 (previously named acidic calponin based on its acidic isoelectric point) (Applegate et al., 1994). Mainly from studies in human and mouse, properties of the three vertebrate calponin isoform genes are summarized in Table 1.

**TABLE 1:** Calponin isoform genes and cell types of expression.

Isoform genes	*CNN1*	*CNN2*	*CNN3*
Protein names	Calponin 1	Calponin 2	Calponin 3
Gene location in human chromosomes	19p13.2-p13.1	19p13.3	1p22-p21
Number of exons	7	7	7
Number of amino acids encoded	297	309	329
Molecular weight of encoded proteins	33.2 kDa	33.7 kDa	36.4 kDa
lsoelectric point of protein products	9.1	7.23	5.84
Cell types of expression	Smooth muscle cells	Smooth muscle cells	Smooth muscle cells
	Mesangial cells	Fibroblasts	Neuronal cells
	Sertoli cells	Epithelial cells	Myoblasts
	Myofibroblasts	Keratocytes	Trophoblasts
		Endothelial cells	B lymphocytes
		B lymphocytes	
		Myeloid blood cells	
		Alveolar cells	
		Carcinoma cells	
		Podocytes	
		Cancer cells	

Calponin 1 is expressed at high levels in smooth muscle and is used as a marker for differentiated smooth muscle cells. The expression level of calponin 1 varies among different types of smooth muscle with high levels in the digestive tract and a very low level in avian trachea (Jin et al., 1996). During mouse embryonic development, calponin 1 protein expression is detectable in E9.5 fetus in the dorsal aorta and tubular heart. Its expression is developmentally upregulated in smooth muscle tissues whereas downregulated in the heart during late fetal development (Samaha et al., 1996). Studies have shown that calponin 1 and calponin 2 are not essential for norepinephrine- and sodium fluoride-induced contractions in rat aortic smooth muscle whereas implying a potential function in modulating contractility (Nigam et al., 1998). The unloaded shortening velocity of thiophosphorylated fibres was significantly faster in the smooth muscle of calponin 1 KO mice than WT control (Matthew et al., 2000). In physiological conditions, calponin does not determine smooth muscle contractile kinetics (Facemire et al., 2000).

In addition to smooth muscle cells, calponin 1 also is expressed in several types of mesenchymal cells, such as glomerular mesangial cells, pancreatic precursor cells, periglomerular myofibroblasts, and Sertoli cells (Zhu et al., 2004). Overexpression of calponin 1 in osteoblast of transgenic mice leads to decreased bone mass by disrupting osteoblast function and promoting osteoclast function (Su et al., 2013). The specific function of calponin 1 in these cell types are not well understood. Similar to smooth muscle actin (Springer et al., 2002), calponin 1 is considered a marker for myofibroblast differentiations which may underlie the significance of its expression in non-muscle cells.

Calponin 2 is expressed in smooth muscle cells. Although structural diversity exists among calponin isoforms, calponin 1 and 2 bind F-actin with similar affinity indicating a conserved mechanism in regulating smooth muscle contractions (Jin et al., 2003). Calponin 2 also expresses in a broad range of non-muscle cell types, including fibroblasts, skin keratinocytes, endothelial cells, lung alveola cells, macrophages, platelets (Hines et al., 2014), osteoblasts, kidney podocytes, breast cancer (Debald et al., 2014), pancreatic cancer (Qiu et al., 2017), and prostate cancer cells (Wu and Jin, 2008; Moazzem Hossain et al., 2014). These diverse cell types can be categorized in three groups: a) cells residing in a tissue environment under high mechanical tension, such as in the wall of hollow organs, b) cells of high rates of proliferation or during development, and c) actively migrating cells, such as fibroblasts and macrophages. Based on calponin’s fundamental function as an inhibitory regulator of myosin ATPase and motor activity (Walsh, 1991), calponin 2 functions in these cell types by regulating motility related process such as cytokinesis (Masuda et al., 1996; Qian et al., 2021), migration (Ulmer et al., 2013; Moazzem Hossain et al., 2014) and phagocytosis (Huang et al., 2008). Calponin 2 expression is relatively restricted to vasculature from 16 to 30 h post-fertilization in a zebrafish embryo study, suggesting calponin 2 is crucial in the migration of endothelial cells during vascular development (Tang et al., 2006). As mechanical tension regulates the transcription of *CNN2* gene and the degradation of calponin 2 protein (Hossain et al., 2005; Hossain et al., 2006; Hossain et al., 2016), the cell type-specific expression and tissue distribution of calponin 2 may reflect a role in sensing and responding to tension signals through adjusting cell motility-based functions (Liu and Jin, 2016a).

Calponin 3 was originally found in rat vascular smooth muscle with expressions also in neural tissues (Applegate et al., 1994). Studies have suggested that calponin 3 is functionally distinct from calponin 1 with unique characteristics even after removal of the acidic tail. For example, although it competes with calponin 1 for binding F-actin, calponin 3 has a weaker inhibitory effect on actomyosin MgATPase (el-Mezgueldi et al., 1996). Different from calponin1, calponin 3 binds F-actin with significant affinity but not microtubules, desmin, tropomyosin, calmodulin, and S100 (Fujii et al., 2002).

Calponin 3 has been studied in several different cell types. A unique function of calponin 3 is that its expression in brain contributes to neural plasticity (Ferhat et al., 2003) and neural tube morphogenesis (Junghans and Herzog, 2018). In addition, calponin 3 has been identified as a negative regulator of the essential cell fusion function of trophoblasts (Shibukawa et al., 2010) and myoblasts (Shibukawa et al., 2013). The inhibitory effects of calponin 3 on myogenesis and myocyte fusion are regulated by the Rho-associated protein kinase (ROCK) signaling pathway (Shibukawa et al., 2013). Another study showed that calponin 3 regulates myoblast proliferation, differentiation and protein synthesis through mTOR pathway (She et al., 2021). Calponin 3 is also found in chondrocytes as a Smad-binding regulator to inhibit bone morphogenic protein (BMP)-mediated transcription (Haag and Aigner, 2007).

Calponin 3 was found critical to coordinated contractility of actin stress fibers (Ciuba et al., 2018). During wound healing, calponin 3 is involved in actin stress fiber remodeling during scar formation and fibrosis (Daimon et al., 2013). Calponin 3 has been shown to regulate cell motility through extracellular signal-regulated kinase (ERK) 1/2-mediated caldesmon phosphorylation (Appel et al., 2010). Expressed in B lymphocytes during early development, calponin 3 functions as a mediator downstream of the precursor B-cell receptor (pre-BCR) in the plasma membrane with Syk-dependent phosphorylation (Flemming et al., 2015).

**FIGURE 1:**
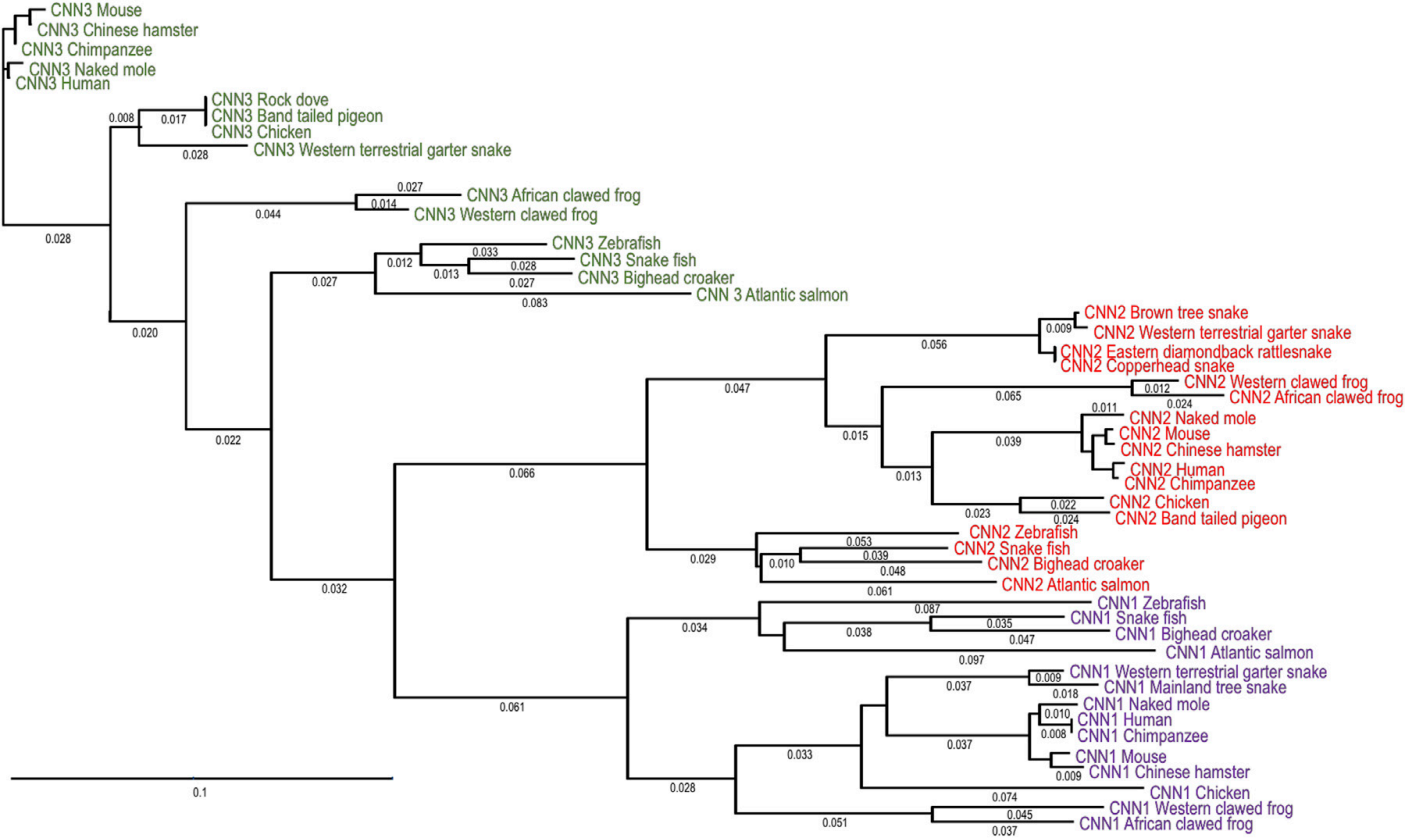
Phylogenetic tree of vertebrate calponin and isoforms. The phylogenetic tree was generated by alignment of amino acid sequences of calponin isoforms in representative vertebrate species with the DNASTAR MegAlign computer software (Lasergene, lnc, Madison, WI) using the Clustal W method. The sequence similarity-derived evolutionary lineages demonstrate that each of the calponin isoforms is conserved in the vertebrate phylum while the three isoforms have significantly diverged during vertebrate evolution. This pattern indicates the adaptation of the calponin isoforms to different cell functions and tissue environments. Calponin isoforms 1, 2, and 3 are marked in purple, red and green, respectively. The degrees of evolutionary divergence are indicated by the lengths of lineage lines. The NCBI database accession numbers of the sequences analyzed are: African clawed frog CNN1, NP_001085014.1; African clawed frog CNN2, ABG49504.1; African clawed frog CNN3, NP_001080482.1; Atlantic salmon CNN1, NP_001139857.1; Atlantic salmon CNN2, NP_001133873.1; Atlantic salmon CNN3, NP_001133337.1; Band tailed pigeon CNN2, OPJ86593.1; Bighead croaker CNN1, TKS92442.1; Bighead croaker CNN2, TKS80042.1; Bighead croaker CNN3, TKS88103.1; Brown tree snake CNN2, JAG68493.1; Chimpanzee CNN1,NP_001267033.1; Chimpanzee CNN2, JAA13388.1; Chimpanzee CNN3, JAA44470.1; Chinese hamster CNN1, EGV96164.1; Chinese hamster CNN2, EGV99480.1; Chinese hamster CNN 3, EGW11625.1; Chicken CNN1, NP_990847.1; Chicken CNN2, NP_001135728.1; Chicken CNN3, NP_001341600.1; Copperhead snake CNN2, JAV51035.1; Eastern Diamondback rattlesnake CNN2, AFJ49586.1; Human CNN1, NP_001290.2; Human CNN2, AAI48265.1; Human CNN3, AAB35752.1; Mainland tiger snake CNN1, XP_026535408.1; Mouse CNN1, AAI38864.1; Mouse CNN2, EDL31614.1; Mouse CNN3, AAH85268.1; Naked mole CNN1, JAN96391.1; Naked mole CNN2, EHB16944.1; Naked mole CNN3, XP_004841280.1; Snakehead fish CNN1, KAF3700611.1; Snakehead fish CNN2, KAF3699176.1; Snakehead fish CNN3, KAF3703402.1; Western terrestrial garter snake CNN1, XP_032066879.1; Western clawed frog CNN1, NP_001015796.1; Western clawed frog CNN2, NP_998841.1; Western clawed frog CNN3, NP_989257.1; Western terrestrial garter snake CNN2, XP_032064388.1; Western terrestrial garter snake CNN3, XP_032074010.1; Zebrafish CNN1, XP_701038.5; Zebrafish CNN2, NP_998514.1; Zebrafish CNN3, NP_956047.1.

Figure 1 show a phylogenetic tree constructed by aligning amino acid sequences of the three calponin isoforms of representative vertebrate species. The evolutionary lineages demonstrate that each of the calponin isoforms is highly conserved in the vertebrate phylum whereas the three isoforms have significantly diverged during evolution. This pattern indicates functional adaptations of the isoforms of calponin to differentiated cellular functions. The data indicate that *Cnn3* emerged earlier than *Cnn1* and *Cnn2* during vertebrate evolution. Consistently, *Cnn3* shows higher degree of divergence among vertebrate classes while *Cnn1* and *Cnn2* are more conserved.

Genetically engineered mouse models provide integrative physiological systems for the study of calponin functions and the significance of tissue-specific high or low level expressions. Experimental data demonstrate that mice with systemic *Cnn1* or *Cnn2* single gene knockout, and *Cnn1, Cnn2* double knockouts are viable and fertile (Takahashi et al., 2000; Feng et al., 2019). Consistently, domestic New Zealand White rabbits have a natural loss of *Cnn2* (Plazyo et al., 2020). In contrast, systemic *Cnn3* knockout resulted in severely defective embryonic neural development with early neonatal lethality (Flemming et al., 2015). With the highly conserved presence in all other vertebrate species studied to date, the lack of calponin 2 in New Zealand White rabbit provides an interesting case of natural *Cnn2* knockout (Plazyo et al., 2020). Although rabbit has been broadly used as a model for the study of human biology and diseases, this unique genetic variation needs to be considered when using rabbit in cell motility-related studies.

Systemic deletion of *Cnn2* in mice decreases postoperative adhesion formation (Hsieh et al., 2021) and causes age progressive proteinuria (Hsieh and Jin, 2022). Cell type-specific calponin knockout mouse models provide more informative experimental systems. B lymphocyte-specific *Cnn3* knockout mice have been used to study B cell development (Springer et al., 2002). Myeloid cell-specific *Cnn2* knockout mice have been used in investigating the functions of calponin 2 in disease models. Mice with myeloid cell-specific deletion of calponin 2 attenuates the development of atherosclerosis (Liu and Jin, 2016b) and reduces inflammatory arthritis through altered macrophage phagocytosis and mobility (Huang et al., 2008; Huang et al., 2016). Supporting this notion, calponin 2 expression in lung residential macrophages is significantly lower than that in peritoneal macrophages corresponding to an adaptation to the maintenance of quiescent state in normal lung with continuing exposure to the external environment (Plazyo et al., 2019). Pathogenic studies showed that systemic deletion but not myeloid cell-specific deletion of calponin 2 prevented the development and progression of calcific aortic valve disease (Plazyo et al., 2018), distinguishing the pathogenesis from that of vascular atherosclerosis.

## 3 Three SM22/Transgelin isoforms in vertebrates

Transgelin was originally identified in 1987 as a 22-kDa protein in chicken gizzard smooth with the name of SM22 (Lees-Miller et al., 1987; Pearlstone et al., 1987). Transgelin/SM22 is one of the most abundant proteins in vertebrate smooth muscles with a high degree of structural similarity to calponin and functions in the stability of actin cytoskeleton similar to that of calponin (Gimona et al., 2003). Represented by human data, three homologous isoforms of transgelin have been identified in vertebrates (Table 2). Transgelin-1 (also named SM22α, p27, WS3-10), transgelin-2 (SM22β) and transgelin-3 (SM22γ, NP25 or NP25) share ∼70% sequence similarity (Liu et al., 2020).

**TABLE 2:** Transgelin isoform genes and cell types of expression.

Isoform genes	*TAGLN*	*TAGLN 2*	*TAGLN 3*
Protein names	Transgelin-1	Transgelin-2	Transgelin-3
Gene location in human chromosomes	11q23.3	11q23.2	3q13.2
Number of exons	5	7	5
Number of amino acids encoded	201	199	199
Molecular weight of encoded proteins	22 kDa	22.39 kDa	22.4 kDa
Isoelectric point of protein products	9	8.4	6.7
Cell types of expression	Smooth muscle cells	Smooth muscle cells	Smooth muscle cells
		Macrophages	Neural cells
		Bone marrow cells	
		pancreatic cells	
		Epithelial cells	
		T lymphocytes	
		Cancer cells	

The conserved structure of transgelin/SM22 consists of an N-terminal CH domain, a calcium binding motif with a helix-loop-helix structure, an actin-binding region, and a single CLIK motif (Fu et al., 2000; Kawasaki and Kretsinger, 2017). Studies have indicated that multiple sites in the C-terminal domain of transgelin/SM22 are required for high affinity actin-binding (Fu et al., 2000). Transgelin/SM22 possesses EF-hand calcium binding sequences with demonstrated Ca^2+^-binding activity (Shishibori et al., 1996).

Transgelin/SM22 is broadly expressed in vascular and visceral smooth muscles and early during smooth muscle differentiation (Gimona et al., 2003). It is also found in fibroblasts and epithelial cells when treated with TGF-β1 to induce myofibroblast differentiation (Elsafadi et al., 2016). Transgelin/SM22 is not required for smooth muscle differentiation but is involved in calcium-independent smooth muscle contraction (Assinder et al., 2009). Recent evidence suggests that transgelin/SM22 acts as a tumour suppressor with diminished expression in prostate (Yang et al., 2007), breast (Sayar et al., 2015; Dvorakova et al., 2016), and colon cancers (Assinder et al., 2009; Liu et al., 2020). This function of transgelin/SM22 may be via suppression of metallomatrix protease-9 (MMP-9) that is upregulated in those types of cancer (Assinder et al., 2009). Transgelin/SM22 has been found in association with remodeling of the actin cytoskeleton and promotes the migration and invasion of cancer stem cells (Liu et al., 2020). Cortical transgelin-2 interacts with light intermediate chain subunit 2 (LIC2)-dynein for maintaining proper length of mitotic spindles, chromosome alignment, spindle orientation and timely anaphase onset (Sharma et al., 2020).

Transgelin-1 has an isoelectric point of 9.0 and is encoded by *TAGLN* gene that is 5.4-kb in size localized on human chromosome 11 at q23.2 (Camoretti-Mercado et al., 1998). Similar to calponin 1, transgelin-1 is primarily and abundantly expressed in smooth muscles (Zhong et al., 2019). Transgelin-1 is used as an early differentiation marker of vertebrate smooth muscle cells (Lawson et al., 1997) and the levels of expression in smooth muscle cells reflect the degree of differentiation (Kato et al., 2019).

Transgelin-2 is also a 22-kDa protein and has an isoelectric point of 8.41 ^68^. Transgelin-2 is encoded by *TAGLN2* gene located on human chromosome 1q23.2 ^74^. Like calponin 2, transgelin-2 is widely expressed in smooth muscle cells (Yin et al., 2018), epithelial cells, stem cells (Kuo et al., 2011), T lymphocytes (Na et al., 2015), bone marrow cells, and pancreatic tissue (Meng et al., 2017). Transgelin-2 localizes to multiple intracellular sites, including the cytoplasm, cell membrane and nucleus. The cellular localizations may vary in pathophysiological conditions, such as epithelial to mesenchymal transition in colorectal cancer (Lin et al., 2009; Zhang et al., 2010; Elsafadi et al., 2020).

Transgelin-3 was first identified and isolated from rat brain in 1994 and named NP25. Encoded by *TAGLN3* gene, it is a protein of 199 amino acids with significant sequence similarity to other transgelin isoforms and calponins (Gimona et al., 2002). The human *TAGLN3* gene located on chromosome 3q13.2. Transgelin-3 has conserved sequences in chicken, rat, mouse, and human, containing an N-terminal CH-domain, an actin binding region and a C-terminal CLIK-sequence (Ren et al., 1994).

**FIGURE 2:**
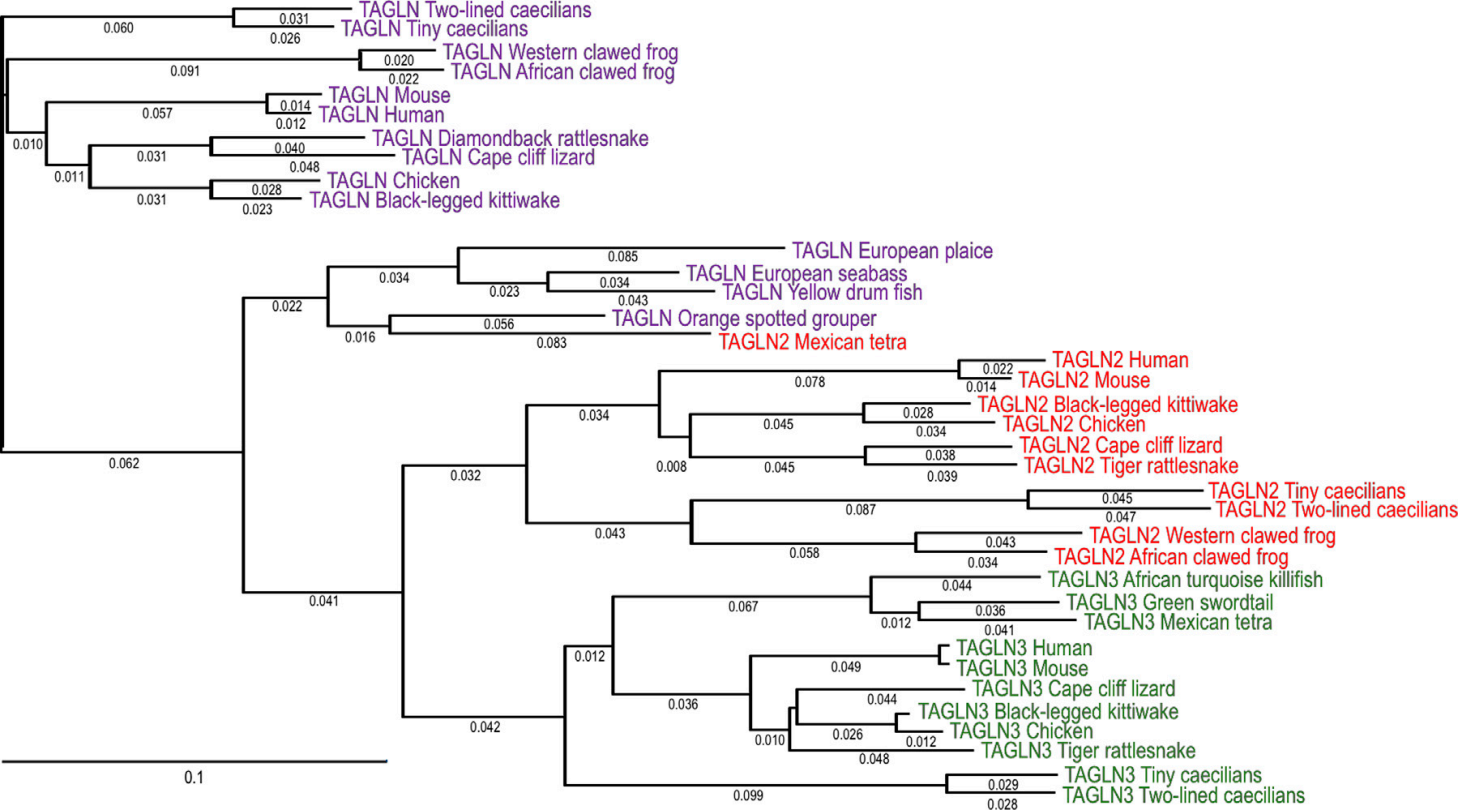
Phylogenetic tree of vertebrate transgelin isoforms. The phylogenetic tree was generated by aligning amino acid sequences of the three transgelin isoforms in representative vertebrate classes including fish, amphibian, reptile, avian, and mammal with the MegAlign computer program (Lasergene; DNASTAR, lnc, Madison, WI) using Clustal W method. The degrees of evolutionary divergence are indicated by the lengths of lineage lines. *TAGLN*, *TAGLN2*, and *TAGLN3* isoforms are marked in purple, red and green, respectively. The NCBI database accession numbers of the sequences analyzed are: African clawed frog TAGLN, NP_001083600.1; African clawed frog TAGLN2 NP_001080783.1; African turquoise killifish TAGLN3, KAF7213900.1; Black-legged kittiwake TAGLN, XP_054078918.1; Black-legged kittiwake TAGLN2, XP_054038817.1; Black-legged kittiwake TAGLN3, XP_054071416.1; Cape cliff lizard TAGLN, XP_053124973.1; Cape cliff lizard TAGLN2, XP_053133737.1; Cape cliff lizard TAGLN3, XP_053167629.1; Chicken TAGLN, AAA48782.1; Chicken TAGLN2, XP_024999416.1; Chicken TAGLN3, XP_040518015.1; Diamondback rattlesnake TAGLN, JAI10881.1; European plaice TAGLN, XP_053298355.1; European seabass TAGLN, XP_051257332.1; Green swordtail TAGLN3, KAF5894368.1; Human TAGLN, NP_001001522.1; Human TAGLN2, KAI4083433.1; Human TAGLN3, KAI2530827.1; Mexican tetra TAGLN2, XP_049332440.1; Mexican tetra TAGLN3, KAG9283353.1; Mouse TAGLN, CAA92941.1; Mouse TAGLN2, NP_848713.1; Mouse TAGLN3, AAH55338.1; Orange spotted grouper TAGLN, ABW04145.1; Tiger rattlesnake TAGLN, 2, XP_039222054.1; Tiger rattlesnake TAGLN3, XP_039220015.1; Tiny caecilian TAGLN, XP_030076731.1; Tiny caecilian TAGLN2, XP_030043052.1; Tiny caecilian TAGLN3, XP_030060064.1; Two-lined caecilian TAGLN, XP_029429188.1; Two-lined caecilian TAGLN2, XP_XP_029436331.1; Two-lined caecilian TAGLN3, XP_029434652.1; Western clawed frog TAGLN, NP_001025579.1; Western clawed frog TAGLN2, NP_989354.1; Yellow drum fish TAGLN, KAG8008337.1.

Figure 2 shows a phylogenetic tree demonstrating the evolution lineages of vertebrate transgelin isoforms. The divergency pattern depicts that each of the transgelin isoforms is conserved across vertebrate species whereas the three isoforms have significantly diverged during vertebrate evolution, which is similar to that of the evolution of calponin isoforms shown in Figure 1. The molecular evolution data indicate that *TAGLN* emerged earlier than *TAGLN2 and TAGLN3* during vertebrate evolution. In the meantime, *TAGLN* has diverged more among vertebrate classes while *TAGLN2* and *TAGLN3* are relatively conserved.

Table 3 shows a comparison of the expression, function and regulation of calponin and transgelin. Calponin and transgelin exhibit similar expression in smooth muscle and non-smooth muscle cells, such as fibroblasts, lymphocytes, neural cells, and cancer cells. Calponin and transgelin both regulate actin cytoskeleton stability and are regulated by mechanical tension. Comparing with that of calponin, more regulatory phosphorylation sites are found in transgelin.

**TABLE 3:** Expression, function and regulation of calponin and transgelin.

Protein	Expression	Function	Mechanism	Regulation
Calponin	Smooth muscle cells	Smooth muscle contractions	Inhibit actin activated myosin motors	PKC phosphorylation
	Fibroblasts, lymphocytes, macrophages	Proliferation, motility, adhesion, migration	Stabilize actin cytoskeleton, tension related mechanoregulation	PKC phosphorylation, Tyrosin phosphorylation, MEKK1 phosphorylation, methylation
	Macrophages	Phagocytosis		
	Neural cells	Cell plasticity		
	Trophoblasts	Cell fusion		
	Prostate cancer cells, ovarian cancer cells	Cancer cell proliferation, migration, adhesion, and metastasis		
Transgelin	Smooth muscle cell	Smooth muscle contractions	Regulate actin cytoskeleton by calponin 2	PKC phosphorylation
	Fibroblasts, lymphocytes, epithelial cells, neural cells	Proliferation, adhesion, migration, phagocytosis	Stabilize actin inhibt actin depolymeralization, tension related mechanotrgulation	PKC phosphorylation, Tyrosin phosphorylation, MEKK1 phosphorylation, methylation, NF-κB, TGF-13β, ERK
	Brease cancer cells, prostate cencer cells, colorectal cancer cells	Cancer cell proliferation, invasion, and metastasis		

## 4 Calponin-like and transgelin-like proteins in invertebrate animals and single cell organisms

Homologs of calponin and transgelin family proteins are found in invertebrates, for example, unc-87 in *Caenorhabditis elegans* (Goetinck and Waterston, 1994) and mp20 in *Drosophila melanogaster* (Ayme-Southgate et al., 1989). While vertebrate muscle tissues are classified as skeletal, cardiac and smooth muscles, muscle tissues of invertebrates are grouped into transverse striates, oblique striates and smooth muscles with variations among species and intermediate types between transverse and oblique striated muscled or between oblique striated and smooth muscles (Plesch, 1977). Same as in vertebrates, calponin is not found in highly differentiated striated muscles of invertebrates. Studies have found calponin expression in invertebrate muscle types structurally similar to vertebrate smooth muscle (Royuela et al., 1997). Calponin was found in the smooth and oblique striated muscles of Helix snail (Royuela et al., 2000) and arm muscles of Antarctic starfish (Meyer-Rochow et al., 2003). During the embryonic development of marine spoon worm *Echiura*, striated and smooth muscle specific genes including calponin-like protein are expressed in specific muscle layers of adult body wall muscles (Han et al., 2020). A study found that freshwater sponge *Ephydatia muelleri* has three transgelin paralogs of which a transgelin-2-like protein was located in contractile bundles in pinacocytes (Colgren and Nichols, 2022).

*Schistosoma japonicum* expresses a 38-kDa protein with substantial structural similarity to vertebrate calponin and expresses exclusively in smooth musculature. This calponin-like protein is located in smooth myofibrils of adult worm in association with myofilaments. It is also present in smooth muscles of the forebody and stratified muscle of the tail. This protein is involved in the contraction of stratified tail muscle, indicating a function similar to smooth muscle calponin in vertebrates (Jones et al., 2001). Calponin-like proteins have been found in regulating octopus muscular contraction and a calponin 2-like isoform was identified in *Octopus bimaculoides* with an interesting function to produce antibacterial peptides against Gram-positive and Gram-negative bacteria (Maselli et al., 2020). Another study also found that calponin expression significantly increased in the honeycomb moth *Galleria mellonella* 24 h after inoculation of *candida* pathogens, implicating a role of calponin in cellular responses to infections (Sheehan and Kavanagh, 2018).

Calponin-like and transgelin-like proteins have been found with more functions in invertebrate organisms besides regulating smooth muscle contraction. During larval-pupal metamorphosis of the cotton bollworm *Helicoverpa armigera*, calponin is expressed at a high level in the metamorphic epidermis and phosphorylated by protein kinase C (Fu et al., 2009). In the embryonic stage, the sea squirt *Ciona* expresses a calponin-transgelin family gene that is crucial to nortocord development (Oonuma et al., 2021). It was shown that non-phosphorylated calponin interacts with ultraspiracles protein to activate the juvenile hormone pathway and antagonize the 20-hydroxyecdysone pathway in initiating insect molting and metamorphosis (Cai et al., 2014). Calponin-like proteins also function in invertebrates during adaption to environmental changes. In the non-calcifying marine worm *Platynereis dumerilii*, expression of a calponin-like gene changed significantly in response to low pH environment (Wage et al., 2016).

**FIGURE 3:**
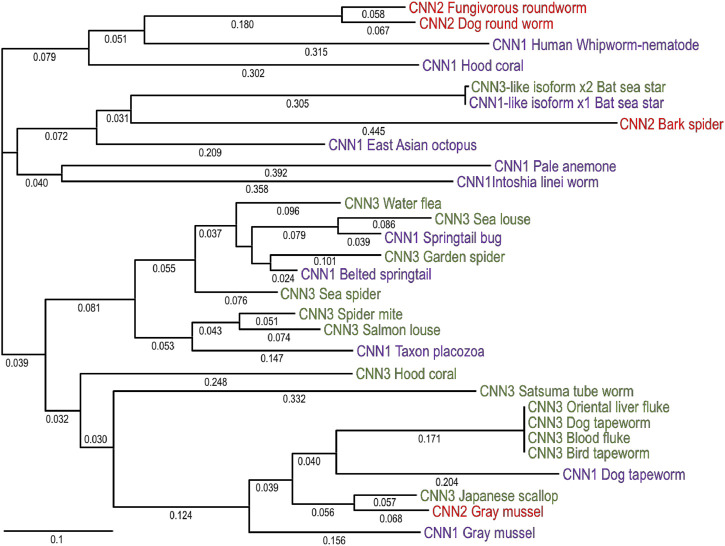
Phylogenetic tree of invertebrate calponin isoforms. The phylogenetic tree of invertebrate calponin was generated by aligning the amino acid sequences of annotated calponin isoforms in the representative invertebrate species with the MegAlign computer program (Lasergene; DNASTAR, lnc, Madison, WI) using Clustal W method. The degrees of evolutionary divergence are indicated by the lengths of lineage lines. Calponin isoforms 1, 2, and 3 are indicated in purple, red and green fonts, respectively. The NCBI database accession numbers of the sequences analyzed are: Bark spider CNN2, GIY95920.1; Bat sea star CNN1, XP_038068192.1; Bat sea star CNN3, XP_038068193.1; Belted springtail CNN1, ODN05277.1; Bird tapeworm CNN3, JAP59453.1; Blood fluke CNN3, XP_012795279.2; Dog round worm CNN2, KHN73181.1; Dog tapeworm CNN1, KAH9282893.1; Dog tapeworm CNN3, EUB56891.1; East Asian octopus CNN1, XP_029648512.1; Fungivorous round worm CNN2, KAH7720906.1; Garden spider CNN3, GBN55272.1; Gray mussel CNN2, APB61452.1; Hood coral CNN1, PFX34187.1; Hood coral CNN3, PFX34186.1; Human whip worm CNN1, CDW60276.1; Intoshia linei worm CNN1, OAF71457.1; Japanese scallop CNN3, OWF41392.1; Oriental liver fluke CNN3, GAA43144.2; Pale anemone CNN1,KXJ22199.1; Taxon placozoa CNN1, RDD43626.1; Water flea CNN3, JAM94970.1; White springtail CNN1, OXA37663.1; Satsuma tube worm CNN3, KAI0207068.1; Salmon louse CNN3, ACO12641.1; Sea louse CNN3, ACO14747.1; Sea spider CNN3, KAG1690965.1; Spider mite CNN3, XP_015791637.1.

Figure 3 shows a phylogenetic tree of invertebrate calponin-like proteins. As invertebrates include 97% of animal species with highly diverged biological backgrounds, the current sequence database indicates that invertebrate calponin is more diverged among species but less distinguishable between isoforms in comparison to that of the three vertebrate isoforms (Figure 1). For example, arthropods represent the most abundant species of animals with over 30 million species (Stork, 2018) but have calponin isoforms closely clustered together despite marine or terrestrial species (Figure 3). This pattern is also shown in the phyla of roundworms and flatworms while their morphological features, organ structures and living environments are very different.

**FIGURE 4:**
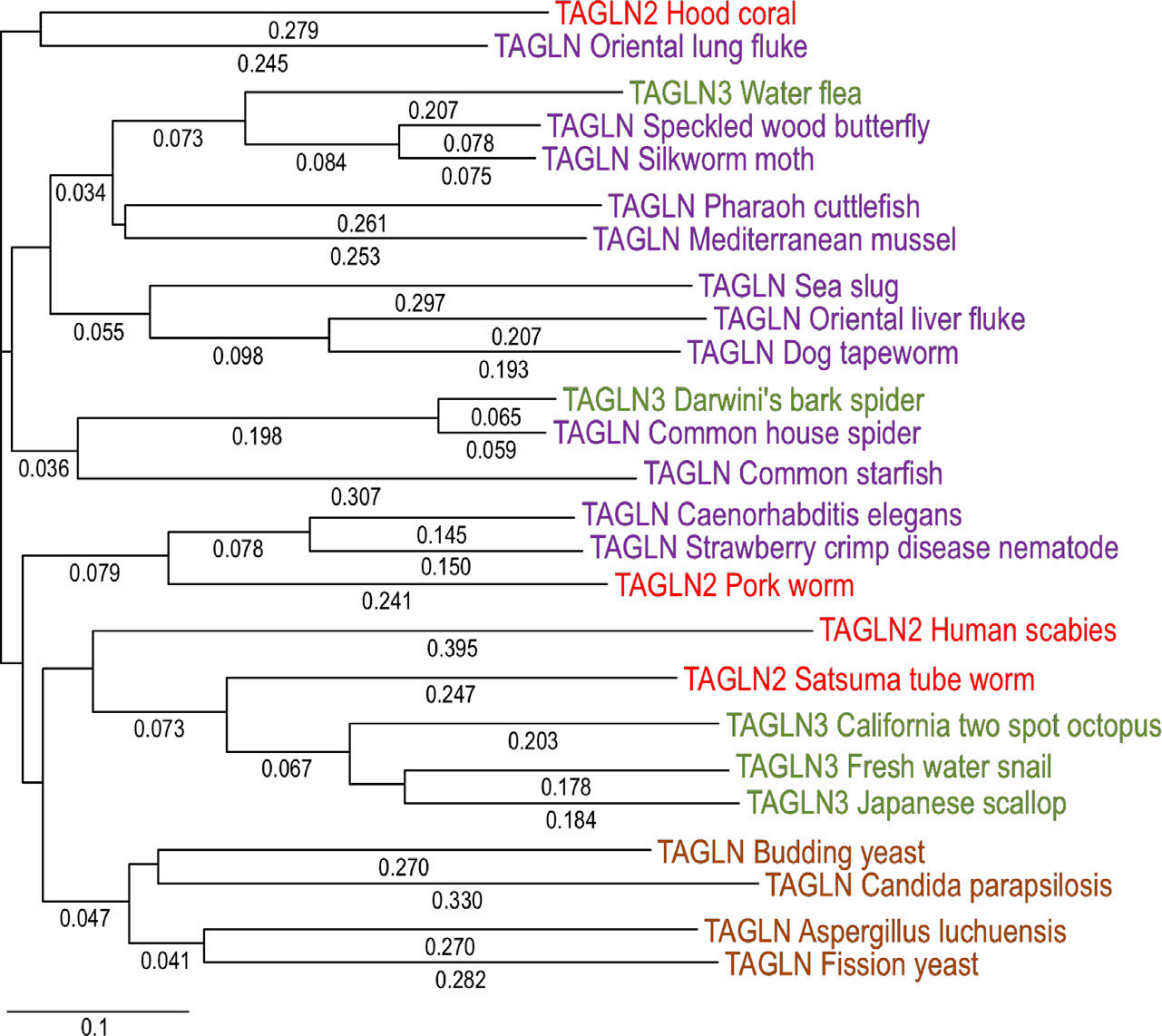
Phylogenetic tree of invertebrate animal and fungal transgelin isoforms. The phylogenetic tree was generated by aligning amino acid sequences of transgelin isoforms of representative invertebrate species with the DNASTAR MegAlign computer software (Lasergene, lnc, Madison, WI) using Clustal W method. Transgelin isoforms 1, 2, and 3 are listed in purple, red and green fonts, respectively. Fungal transgelins are highlighted in brown font. The NCBI database accession numbers of the sequences are: California two spot octopus TAGLN3, XP_014777580.1; *Caenorhabditis elegans* TAGLN, NP_493713.2; Common house spider TAGLN, LAA01862.1; Common starfish TAGLN, QAA95982.1; Darwini’s bark spider TAGLN3, GIY80101.1; Dog tapeworm TAGLN, KAH9282095.1; Oriental liver fluke TAGLN, KAG5444608.1; Freshwater snail TAGLN3, KAH9519225.1; Hood coral TAGLN2, PFX34185.1; Human scabies TAGLN2, KAI7688587.1; Japanese scallop TAGLN3, OWF50751.1; Mediterranean mussel TAGLN, VDI10566.1; Oriental lung fluke TAGLN, KAA3679206.1; Pharaoh cuttlefish TAGLN, CAE1295289.1; Pork worm TAGLN2, KRY33709.1; Satsuma tube worm TAGLN2, KAI0231824.1; Sea slug TAGLN, GFO13790.1; Silkworm moth TAGLN, ABF51271.1; Speckled wood butterfly TAGLN, JAA92459.1; Strawberry crimp disease nematode TAGLN, KAI6193121.1; Water flea TAGLN3, JAJ10604.1; Aspergillus luchuensis TAGLN, GAT26957.1; Budding yeast TAGLN, KAF4004496.1; *Candida* parapsilosis TAGLN, KAI5906036.1; Fission yeast TAGLN, O14185.1.

Similar patterns are found in the phylogeny of invertebrate transgelin and isoforms as shown in Figure 4. Transgelin-like proteins have been identified in fungi, for example, *STG*1, a protein of 174 amino acids in fission yeast (Nakano et al., 2005) and *SCP*1, a protein of 200 amino acids in budding yeast (Goodman et al., 2003; Winder et al., 2003). The transgelin-like proteins appear evolutionarily conserved in the kingdom of fungi (Figure 4).

## 5 Structure-function relationships of calponin and transgelin

While the three vertebrate calponin isoforms have clearly diverged during evolution, each of the isoforms is conserved across fish, amphibian, reptile, avian, and mammalian classes (Figure 1). Although each of the vertebrate calponin isoforms may have evolved to execute different physiological functions based on their cell type-specific distributions, their core structures are conserved, reflecting conserved core functions (Liu and Jin, 2016a).

**FIGURE 5:**
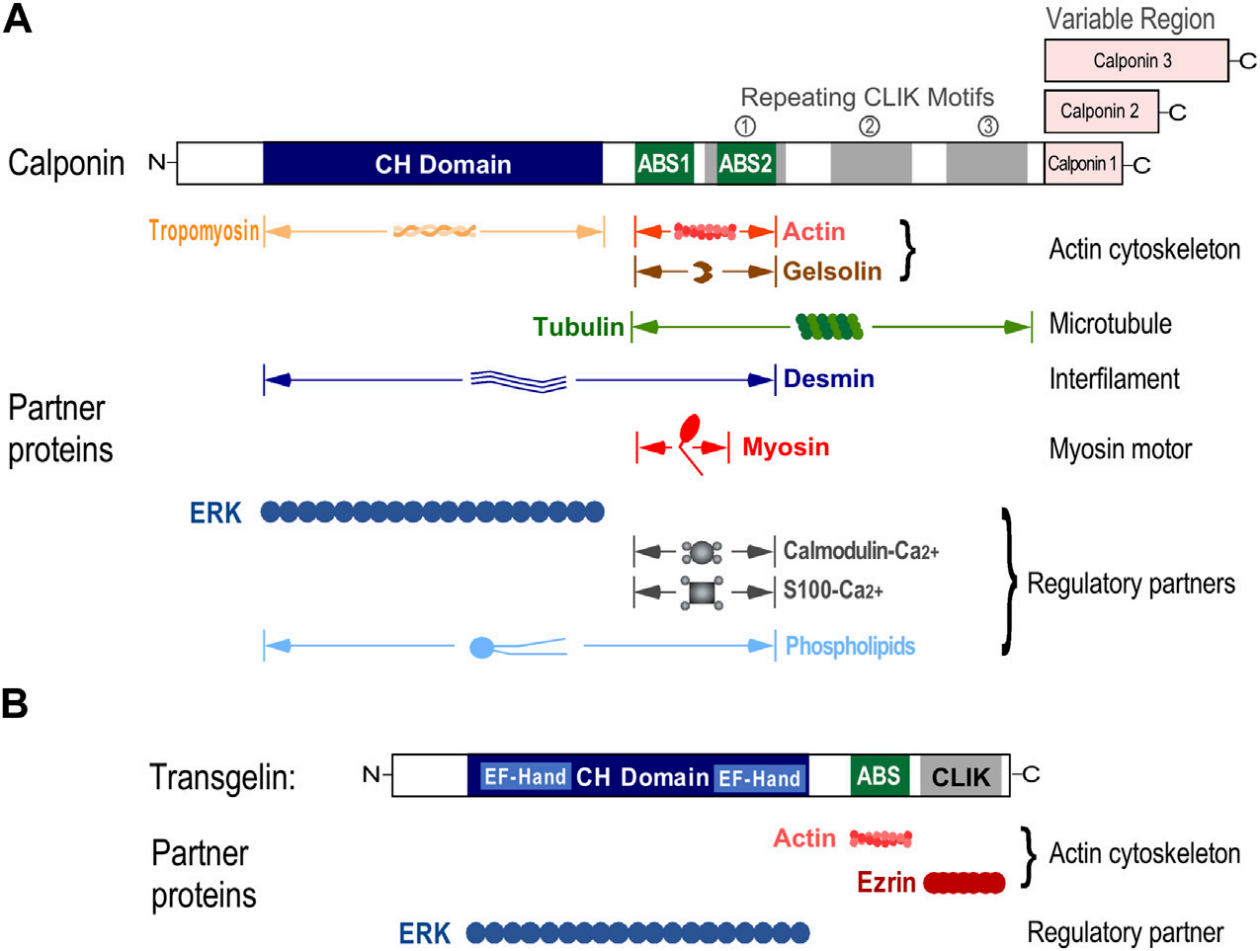
Cytoskeleton and regulatory partners of calponin and transgelin. **(A)** Multiple cytoskeleton and regulatory proteins have been identified to interact with calponin. Calponin binds actin filaments through two actin-binding sites (ABS), tropomyosin via the N-terminal calponin homology (CH) domain, and gelsolin via the actin binding sites. Calponin interacts with microtubules through the actin-binding sites and the repeating motifs whereas with desmin through the region of CH domain and the actin-binding sites. Residues 144–182 of calponin interact with myosin. Ca^2+^-calmodulin and Ca^2+^-S100 bind calponin at the actin-binding sites, which releases the inhibitory effect on myosin MgATPase. An N-terminal fragment of calponin was reported to interact with phospholipids and the CH domain overlaps with an ERK binding region. **(B)** In transgelin, the CH domain binds ERK, the ABS domain binds F-actin, and the C-terminal calponin like (CLIK) segment binds ezrin. Transgelin interacts with ezrin through its CLIK domain. Two EF-hand Ca binding motifs are found in the CH domain of transgelin, suggesting a possible regulation via calcium signaling.

As an actin filament-associated regulatory protein, the roles of calponin in cytoskeleton and cell motility functions have been extensively investigated (Liu and Jin, 2016a). Biochemical studies of calponin 1 in the context of smooth muscle regulation have shown that in addition to binding to actin and crosslinking actin filaments, calponin interacts with other cytoskeleton and related proteins such as tropomyosin, myosin, tubulin, desmin, gelsolin, Ca^2+^-calmodulin, Ca^2+^-S100, and phospholipids. Calponin may also act as a cross-linking protein between desmin filaments as well as intermediate filaments, microfilaments and microtubules in smooth muscle cells (Fujii et al., 2000). The thin filament inhibitory regulation of calponin on actomyosin ATPase and motor function has been correlated with increased isometric force generation, increased actin filament binding to myosin but decreased moving velocity of actin filaments over thiophosphorylated smooth muscle myosin in in vitro motility assay (Haeberle, 1994). The interaction of calponin and caldesmon with phospholipids may play a role in regulating the dynamics of cytoskeleton (Gusev, 2001). The F-actin severing protein gelsolin may form high-affinity complexes with calponin 1 and calponin 3 through its actin binding sites as gelsolin does not interact with calponin when calponin is bound to F-actin (Ferjani et al., 2006). The known cytoskeleton and regulatory partners of calponin and transgelin are summarized in Figure 5.

The function of calponin in non-muscle cells was mostly studied with calponin 2. Calponin 2 have been shown to stabilize the actin cytoskeleton (Hossain et al., 2005) and regulate cellular activities including proliferation (Hossain et al., 2003; Moazzem Hossain et al., 2014), adhesion (Moazzem Hossain et al., 2014; Hsieh et al., 2021), migration (Ulmer et al., 2013), cell traction force (Hossain et al., 2016), myofibroblast differentiation (Plazyo et al., 2018), and phagocytosis (Huang et al., 2008; Huang et al., 2016). Calponin and transgelin have been found to closely related to cancer metastasis (Verone et al., 2013; Liu et al., 2020) and autoimmune diseases (Huang et al., 2016; Yin et al., 2018). In invertebrates, transgelin is related to calcium crystal formation in mollusks critical to shell formation. A study found that calponin is crucial in early cellular and humoral response to infection in the honeycomb moth *Galleria mellonella* (Sheehan and Kavanagh, 2018).

**FIGURE 6:**
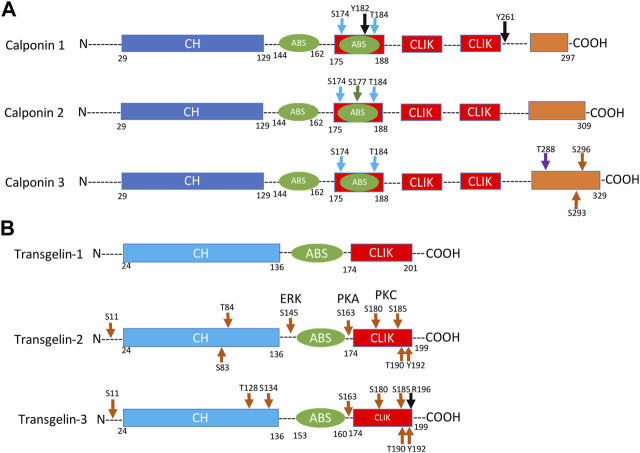
Structural comparison of vertebrate calponin and transgelin isoforms. **(A)** The primary structural comparison of three calponin isoforms shows the N-terminal calponin homology (CH) domain, the two actin-binding sites (ABS), the three calponin like repeat (CLIK) motifs, and the C-terminal variable region that constitutes the main differences between the isoforms. Conserved PKC phosphorylation sites in all three isoforms include Ser_175_ and Thr_184_ (blue arrow) in the second actin-binding site overlapping with the first CLIK motif. Calponin 1 has two tyrosine phosphorylation sites at Tyr_261_ and Tyr_182_ (black arrow). Calponin 2 has a potentially phosphorylation site at Ser_177_ (green arrow). Calponin 3 has two ROCK phosphorylation sites at Ser_293_ and Ser_296_ as well as an MEKK1 phosphorylation site at Thr_288_ in the C-terminal region. **(B)** The structure of human transgelin consists of three regions: An N-terminal CH domain, an ABS, and a C-terminal CLIK region, which resembling a mini-calponin lacking the C-terminal variable tail. Phosphorylation sites have been found in transgelin-2 at Ser_11_, Ser83, Thr_84_, Ser_145_, Ser_163_, Ser_180_, Ser_185_, Thr_190_, and Tyr_192_. In transgelin-3, phosphorylation sites have been identified at Ser_11_, Thr_128_, Ser_134_, Ser_163_, Ser_180_, Ser_185_, and Thr_190_, and a methylation site at Arg_196_.

The primary structure of vertebrate calponin is highly conserved. While there are some variations among species, we may focus on the structure of human calponin isoforms to summarize the structure-function relationships. Figure 6A aligns the primary structural maps of three vertebrate calponin isoforms. The three calponin isoforms share the conserved N-terminal calponin homology (CH) domain, the middle region containing two actin-binding sites (ABS) and the three calponin like (CLIK) repeating motifs. The C-terminal segment of calponin is a variable region that constitutes the main structural differences between the three isoforms.

The actomyosin ATPase inhibitory site locates in the segment of amino acids 145–182 which also binds F-actin and calmodulin (Fattoum et al., 2003). Mapped in calponin 1 studies, two microtubule-binding sites were identified, one related to the inhibitory site in the segment 145–182 and the other specific for microtubule locates in the segment 183–292 (Fattoum et al., 2003). A recent study reported a heat shock protein (hsp) 90-binding site in the N-terminal (residues 7–144) portion of calponin with a function in regulating the formation of actin bundles (Ma et al., 2000).

Demonstrated in smooth muscle studies, calponin 1 functions as an inhibitor of actin-activated myosin ATPase and motor function under the regulation of Ca^2+^-calmodulin or phosphorylation (Walsh, 1991; Hossain et al., 2016). A Ca^2+^-calmodulin-binding site is mapped to the segment of amino acids 52–144 (Mezgueldi et al., 1992) (Figure 6A). Protein kinase C (PKC) phosphorylation sites likely common for all three isoforms are located at Ser_175_ and Thr_184_ (blue arrow) in the second actin-binding site in the first CLIK motif (Figure 6A). In addition to the PKC phosphorylation sites, two tyrosine phosphorylation sites were identified in calponin 1 at Tyr261 and Tyr_182_ (black arrow in Figure 6A). Calponin 2 has a potentially additional phosphorylatable serine at position 177 near the PKC-phosphorylated Ser_175_. Calponin 3 has two ROCK phosphorylation sites at Ser_293_ and probably Ser296 (Shibukawa et al., 2013) as well as an MEKK1 phosphorylation site at Thr_288_ in the C-terminal region (Hirata et al., 2016) (Figure 6A).

The linear structure maps in Figure 6B demonstrate that the conserved structure of human transgelin isoforms consisting of three elements: An N-terminal CH domain, an ABS and a C-terminal CLIK segment. The CH domain of transgelin has been shown to bind ERK whereas its ABS domain binds actin and C-terminal CLIK domain binds ezrin (Yin et al., 2019). Two EF-hand Ca^2+^-binding motifs are found in the CH domain of transgelin, suggesting a possible role of transgelin in Ca^2+^-mediated cell regulations (Matsui et al., 2018). Multiple potential phosphorylation sites are found in transgelin-2 at Ser_11_, Ser_83_, Thr_84_, Ser_145_, Ser_163_, Ser_180_, Ser_185_, Thr_190_, and Tyr_192_ (Zhou et al., 2013; Jang et al., 2015). Transgelin-3 contains implicated phosphorylation sites at Ser_11_, Thr_128_, Ser_134_, Ser_163_, Ser_180_, Ser_185_ and Thr_190_, and a methylation site at Arg_196_ (Wu et al., 2021).

Transgelin and calponin both contain a CH domain (Li et al., 2008). Whereas calponin has two actin-binding sites, transgelin has only one homologous to the first actin-binding site of calponin (Fu et al., 2000), which shares properties of other actin-binding proteins (Fu et al., 2000). Similarly, transgelin has only one CLIK motif versus the two in calponins. In contrast to the three calponin isoforms, transgelin does not have the isoform-specific C-terminal variable region (Figure 6). As transgelin contains all of the key structures of calponin without repeats or variable region, it can be considered as a minimal version of calponin with a concise structure for the core functions of calponin-transgelin family proteins.

The calponin homology (CH) domain is a sequence motif of around 100 amino acids. It was first identified in the N-terminal region of calponin 1 spanning amino acid residues 29–129 (Castresana and Saraste, 1995; Gimona et al., 2002). CH domain has since been identified in numerous actin-binding proteins. The CH domain-containing proteins are in three major groups with one to four of CH domains (Korenbaum and Rivero, 2002). Grouped in the calponin family (Korenbaum and Rivero, 2002), calponin and transgelin are single CH domain proteins. Proteins with multiple CH domains on the other hand represent a different module of proteins (Stradal et al., 1998).

No definitive biochemical function has been identified for the CH domain in calponin or transgelin. The CH domain is not responsible for binding F-actin nor regulating the actin-binding (Galkin et al., 2006). The CH domain in calponin was reported to bind to extracellular regulated kinase (ERK) (Leinweber et al., 1999) and calponin was coimmunoprecipitated with mitogen-activated protein kinase (MAPK) (Menice et al., 1997), suggesting that calponin may serve as adaptor protein in the ERK signaling cascades of smooth muscle and non-muscle cells.

**FIGURE 7:**
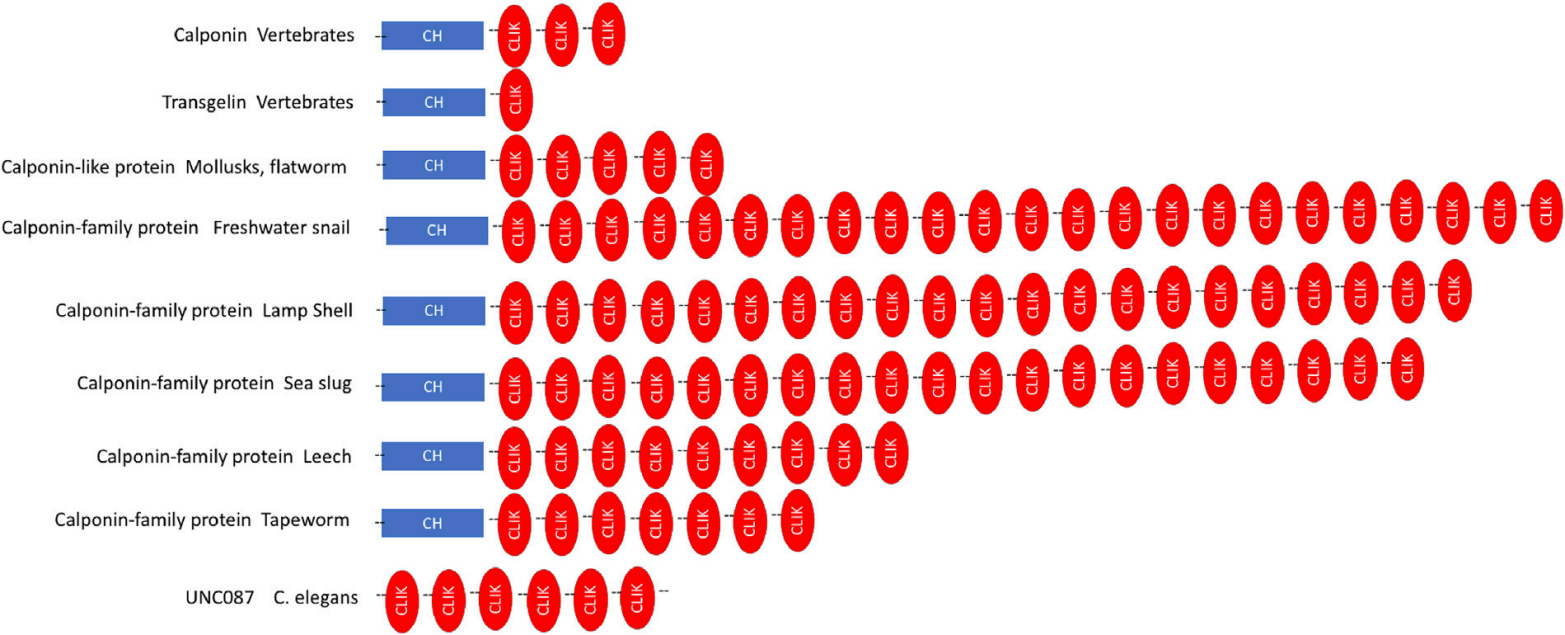
Linear structural maps of invertebrate calponin-like and transgelin-like proteins. The structures of invertebrate calponin-transgelin family proteins are compared. The blue box indicates the N-terminal calponin homology (CH) domain and the red ellipses are calponin like (CLIK) motifs. The invertebrate calponin-transgelin family proteins contain a CH domain and highly diverse numbers of CLIK repeats whereas the calponin-related protein unc-87 of *Caenorhabditis elegans* does not have the CH domain (Ono, 2021).

The structural variations of invertebrate calponin-like and transgelin-like proteins are significantly larger than that in vertebrate homologs. Calponin-like protein in invertebrates was first identified in mussels (Sirenko et al., 2016) as a major regulatory protein for actin and tropomyosin related cellular functions, which contains a CH domain and five CLIK repeats (Figure 7). Several other invertebrate calponin-like proteins have since been reported containing a CH domain and a various number of CLIK repeats, ranging from 7 in tapeworm to 23 in mollusks (Ono, 2021). The calponin-related protein unc-87 of *Caenorhabditis elegans* consists of seven CLIK repeats in the absence of the CH domain (Ono, 2021).

## 6 Regulation of calponin and transgelin gene expression and function

The specific expression of transgelin-1/SM22α in differentiated smooth muscle is similar to that of calponin 1 (Duband et al., 1993) whereas its regulation of actin cytoskeleton in fibroblasts is similar to that of calponin 2 in fibroblast (Liu et al., 2017) and cancer cells (Thompson et al., 2012). Both calponin 2 and transgelin-2 are regulators for cell division (Moazzem Hossain et al., 2014; Zhang et al., 2018). Cell cycle-regulated degradation of calponin 2 is critical to the completion of cytokinesis (Qian et al., 2021).

In addition to modgulating smooth muscle contractility, calponin and transgelin regulate the structure and function of actin filaments in multiple types of non-muscle cells for various cellular activities. Therefore, the expressions of calponin and transgelin need to be regulated in different physiological processes. Several studies have investigated the regulation of calponin gene expression at transcriptional and posttranslational levels. During quail and chicken embryonic development, calponin 1 is expressed in muscularizing tissue of endocardial cushion while other smooth muscle-specific genes such as smooth muscle actin and caldesmon are not expressed (Moralez et al., 2006). Calponin and transgelin play a major role in tumorigenesis and cancer metastasis. The first CLIK repeat of calponin 1 showed tumor suppression effect in in vitro proliferation, cell motility, and soft agar invasion assays (Yamane et al., 2015). Transgelin binds poly ADP-ribose polymerase 1 (PARP1) and regulates the downstream Rho signaling pathway in the metastasis of colon cancer (Lew et al., 2020). *TAGLN2*-annexin A2 (*ANXA2*) interaction and NF-kappa B signaling pathway are related to the invasion and metastasis of hepatocellular carcinoma (Shi et al., 2020). Trangelin-2 regulates T cell activation by stabilizing the actin cytoskeleton at the immunological synapse, which is crucial for cytokine production and cytotoxic effector function (Na et al., 2015).

Extensive studies have investigated the phosphorylation regulation of calponin. Studies of calponin 1 have identified two primary PKC phosphorylation sites at residues Ser_175_ and Thr_184_ in the second actin-binding site and the first CLIK motif. These sites are highly conserved in the three calponin isoforms (Figure 6A). Ser_175_ is the principal substrate of PKC and calmodulin-dependent protein kinase, which regulates the binding of calponin to actin and the inhibition of myosin cross-bridge cycling rate (Tang et al., 1996). Phosphorylation of Ser_175_ also alters the structural flexibility of calponin to increase antibody epitope accessibility (Jin et al., 2000). PKC signaling of calponin regulates redistribution of calponin in the cell. Studies have shown that calponin 1 undergoes a PKC agonist-induced translocation from the central contractile filaments to the membranous cortical area (Parker et al., 1994). Through phosphorylation at Ser_175_, calponin may serve as an adaptor protein linking the PKC signaling cascade to the ERK cascade (Leinweber et al., 2000). In vascular smooth muscle cells, calponin may activate PKC autophosphorylation in a lipid-independent manner (Leinweber et al., 2000; Kim et al., 2013). The function of calponin is also regulated by dephosphorylation catalyzed by type 2B protein phosphatase (Ichikawa et al., 1993; Fraser and Walsh, 1995).

In calponin 1, both Tyr_261_ and Tyr_182_ are phosphorylation sites for tyrosine kinase (Abouzaglou et al., 2004). Tyr_261_ of calponin 3 has also been shown to be phosphorylated by tyrosine kinase (Abouzaglou et al., 2004). ROCK phosphorylates calponin 3 at Ser_293_ and Ser_296_ in association with actin cytoskeleton and accelerates plasma membrane fusion. ROCK phosphorylation diminishes the inhibitory effect of calponin 3 on muscle cell differentiation and fusion (Shibukawa et al., 2013). Mitogen activated protein kinase-1 (MEKK1) phosphorylates calponin 3 at Thr_288_ to tune cell contractility and increase traction force (Hirata et al., 2016). The complex of phosphorylation sites involving different kinases may reflect the regulation of broad functions by calponin and transgelin in diverse types of cells in physiological and pathological conditions, which remains to be investigated.

The gene expression and function of calponin and transgelin are regulated by mechanical tension. This was first demonstrated by culturing human skin keratinocytes on gel substrates of various stiffness to show that higher stiffness-generated higher cytoskeleton tension upregulates the expression of *Cnn2* gene (Hossain et al., 2005). The mechanical tension-regulation of calponin 2 also acts posttranslational as shown by a low tension-induced degradation in lung alveolar cells (Hossain et al., 2006). An average static tension rather than dynamic tension dependent expression of *Cnn2* is demonstrated in cells cultured under various regimes of cyclic stretching (Hossain et al., 2006). The mechanoregulation of *Cnn2* gene promoter involves the Notch signaling pathway (Jiang et al., 2014). A study has demonstrated that the gene expression and protein degradation of transgelin-1 in smooth muscle are also regulated by mechanical tension similarly to that of calponin 2 (Liu et al., 2017). Consistently, collagen represses canonical Notch signaling and regulates the downstream gene expression of both calponin and transgelin (Zhang et al., 2013). Studies further showed transgelin-2 participates in the progression of colorectal cancer through interacting with CD44 to regulate the Notch1 signaling pathway (Ding et al., 2022). These findings implicate functional and mechanistic similarities between the regulation and function of calponin and transgelin.

## 7 Summary and perspectives

**FIGURE 8:**
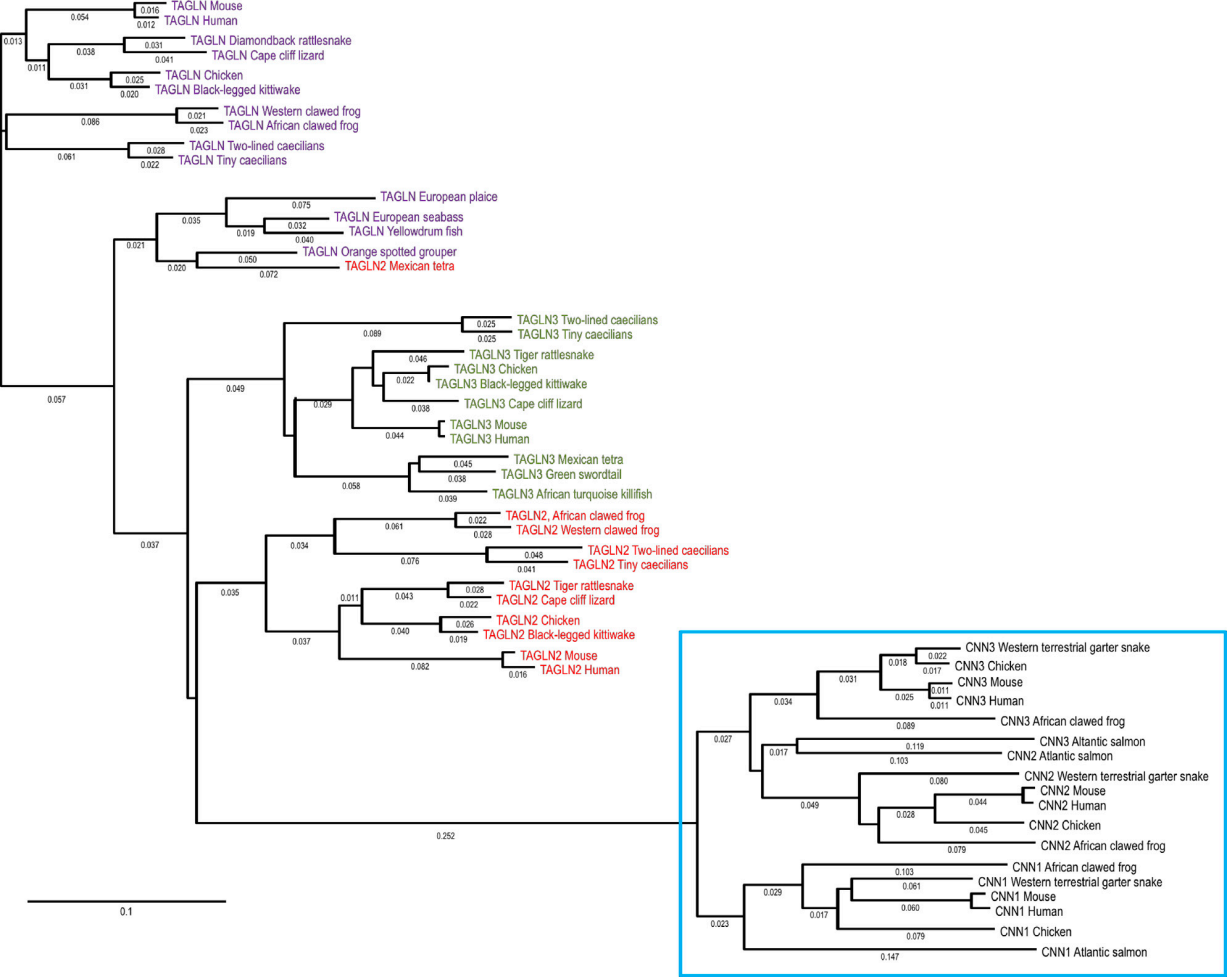
Phylogeny of the calponin-transgelin superfamily genes in vertebrates. The combined phylogenetic tree of vertebrate calponin and transgelin isoforms was generated by aligning amino acid sequences of representative species with the DNASTAR MegAlign computer software (Lasergene, lnc, Madison, WI) using Clustal W method. Transgelin isoforms 1, 2, and 3 are marked in purple, red and green fonts, respectively. The vertebrate calponin isoforms 1, 2, and 3 are clustered in the blue box. The phylogenetic lineages demonstrate that calponin and transgelin diverged early during vertebrate evolution and transgelin-1 emerged before transgelin-2, transgelin-3 and the three calponin isoforms. The NCBI database accession numbers of the sequences are listed in the legends of Figures 1, 2.

Based on the structural similarity of vertebrate calponin and transgelin, the combined phylogenetic analysis in Figure 8 demonstrates that these two proteins are evolutionary homologous. The data clearly support that calponin and transgelin are members of a super gene family with conserved functional similarities. The phylogenetic lineages of vertebrate calponin and transgelin isoforms also show that transgelin isoforms are diverged earlier but less distinguished from each other than that of the calponin isoforms. Therefore, while each of the clearly diverged three calponin isoforms is highly conserved across vertebrate phylum to represent specialized functions, the transgelin isoforms especially the earliest emerged transgelin-1/SM22α (Figure 8) may represent the ancestral form and core structures (Figure 6B) of this superfamily of cytoskeleton regulatory proteins in vertebrates.

**FIGURE 9:**
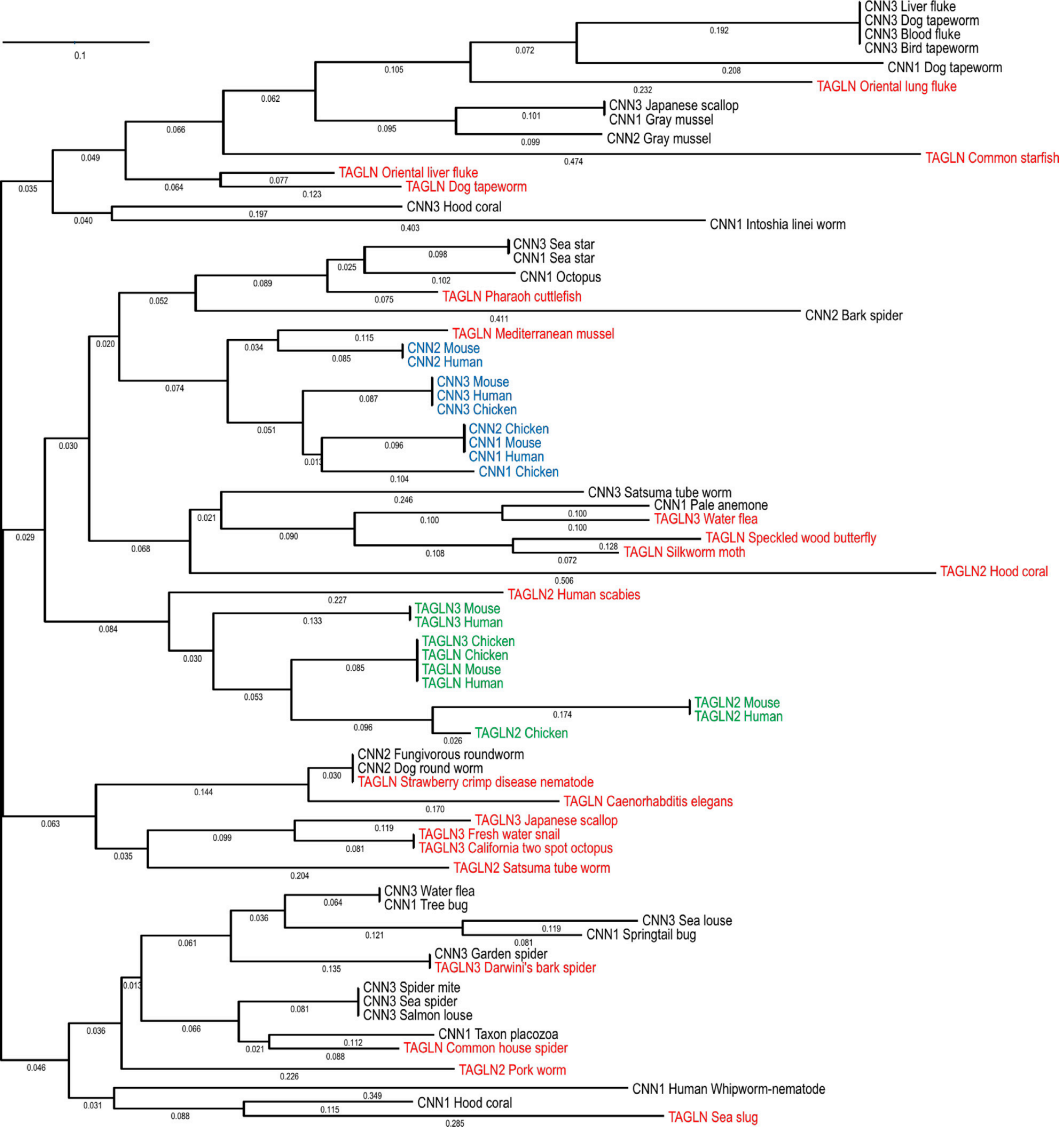
Overall phylogeny of calponin-transgelin genes in vertebrate and invertebrate animals. The combined phylogenetic tree of vertebrate and invertebrate calponin and transgelin was generated by aligning amino acid sequences of representative species with the DNASTAR MegAlign computer software (Lasergene, lnc, Madison, WI) using Clustal W method. Vertebrate calponin isoforms are in blue font, invertebrate calponins are in black font, vertebrate transgelins are in green font and invertebrate transgelins are in red font. The NCBI database accession numbers of the sequences are in the legends of Figures 1–4. The evolutionary lineages show that while calponin and transgelin have evolved into distinct and conserved isoforms in vertebrates, the invertebrate homologs are significantly less differentiated.

To further demonstrate the overall phylogeny of calponin-transgelin superfamily proteins, the combined phylogenetic analysis of calponin and transgelin isoform genes in vertebrate and invertebrate animals in Figure 9 confirms that while calponin and transgelin both emerged early in animal evolution indicating fundamental biological functions, transgelin was the ancestor representing prototype structure and functions. The clearly diverged calponin isoforms in vertebrates reflect specialized functions whereas the less distinguishable transgelin isoforms implicate likely housekeeping functions as demonstrated in recent literature (Meng et al., 2017; Jo et al., 2018).

In conclusion, calponins and transgelins are members of an evolutionarily homologous superfamily of actin cytoskeleton regulatory proteins. The evolutionary lineages demonstrate their structural and functional similarities. Cell type specific isoforms of calponin and transgelin have evolved in vertebrates with functions in regulating cell proliferation, adhesion, migration, phagocytosis, cell fusion, and inflammatory and fibrotic responses. With a growing body of evidence shows major functional importance of calponin and transgelin, there are major knowledge gaps that limit our understanding of the biological function and regulation of calponin and transgelin. Continued and extended research is needed to further establish the physiological and pathological significances of calponin and transgelin in human health and diseases.

## References

[B1] Abouzaglou J. Benistant C. Gimona M. Roustan C. Kassab R. Fattoum A. (2004). Tyrosine phosphorylation of calponins. Inhibition of the interaction with F-actin. Eur. J. Biochem. 271 (13), 2615–2623. 10.1111/j.1432-1033.2004.04190.x 15206927

[B2] Appel S. Allen P. G. Vetterkind S. Jin J. P. Morgan K. G. (2010). h3/Acidic calponin: an actin-binding protein that controls extracellular signal-regulated kinase 1/2 activity in nonmuscle cells. Mol. Biol. Cell 21 (8), 1409–1422. 10.1091/mbc.e09-06-0451 20181831 PMC2854098

[B3] Applegate D. Feng W. Green R. S. Taubman M. B. (1994). Cloning and expression of a novel acidic calponin isoform from rat aortic vascular smooth muscle. J. Biol. Chem. 269 (14), 10683–10690. 10.1016/s0021-9258(17)34113-3 8144658

[B4] Assinder S. J. Stanton J. A. Prasad P. D. (2009). Transgelin: An actin-binding protein and tumour suppressor. Int. J. Biochem. Cell Biol. 41 (3), 482–486. 10.1016/j.biocel.2008.02.011 18378184

[B5] Ayme-Southgate A. Lasko P. French C. Pardue M. L. (1989). Characterization of the gene for mp20: A Drosophila muscle protein that is not found in asynchronous oscillatory flight muscle. J. Cell Biol. 108 (2), 521–531. 10.1083/jcb.108.2.521 2537318 PMC2115408

[B6] Cai M. J. Liu W. Pei X. Y. Li X. R. He H. J. Wang J. X. (2014). Juvenile hormone prevents 20-hydroxyecdysone-induced metamorphosis by regulating the phosphorylation of a newly identified broad protein. J. Biol. Chem. 289 (38), 26630–26641. 10.1074/jbc.M114.581876 25096576 PMC4176204

[B7] Camoretti-Mercado B. Forsythe S. M. LeBeau M. M. Espinosa R. Vieira J. E. Halayko A. J. (1998). Expression and cytogenetic localization of the human SM22 gene (TAGLN). Genomics 49 (3), 452–457. 10.1006/geno.1998.5267 9615232

[B8] Castresana J. Saraste M. (1995). Does Vav bind to F-actin through a CH domain? FEBS Lett. 374 (2), 149–151. 10.1016/0014-5793(95)01098-y 7589522

[B9] Ciuba K. Hawkes W. Tojkander S. Kogan K. Engel U. Iskratsch T. (2018). Calponin-3 is critical for coordinated contractility of actin stress fibers. Sci. Rep. 8 (1), 17670. 10.1038/s41598-018-35948-6 30518778 PMC6281606

[B10] Colgren J. Nichols S. A. (2022). MRTF specifies a muscle-like contractile module in Porifera. Nat. Commun. 13 (1), 4134. 10.1038/s41467-022-31756-9 35840552 PMC9287330

[B11] Daimon E. Shibukawa Y. Wada Y. (2013). Calponin 3 regulates stress fiber formation in dermal fibroblasts during wound healing. Arch. Dermatol Res. 305 (7), 571–584. 10.1007/s00403-013-1343-8 23545751

[B12] Debald M. Jin J. P. Linke A. Walgenbach K. J. Rauch P. Zellmer A. (2014). Calponin-h2: A potential serum marker for the early detection of human breast cancer? Tumour Biol. 35 (11), 11121–11127. 10.1007/s13277-014-2419-6 25099617

[B13] Ding R. Li G. Yao Y. Zhang L. Zhang X. Li J. (2022). Transgelin-2 interacts with CD44 to regulate Notch1 signaling pathway and participates in colorectal cancer proliferation and migration. J. Physiol. Biochem. 78 (1), 99–108. 10.1007/s13105-021-00843-8 34553339

[B14] Dobrzhanskaya A. V. Vyatchin I. G. Lazarev S. S. Matusovsky O. S. Shelud'ko N. S. (2013). Molluscan smooth catch muscle contains calponin but not caldesmon. J. Muscle Res. Cell Motil. 34 (1), 23–33. 10.1007/s10974-012-9329-2 23081709

[B15] Duband J. L. Gimona M. Scatena M. Sartore S. Small J. V. (1993). Calponin and SM 22 as differentiation markers of smooth muscle: Spatiotemporal distribution during avian embryonic development. Differentiation 55 (1), 1–11. 10.1111/j.1432-0436.1993.tb00027.x 8299876

[B16] Dvorakova M. Jerabkova J. Prochazkova I. Lenco J. Nenutil R. Bouchal P. (2016). Transgelin is upregulated in stromal cells of lymph node positive breast cancer. J. Proteomics 132, 103–111. 10.1016/j.jprot.2015.11.025 26639304

[B17] el-Mezgueldi M. Strasser P. Fattoum A. Gimona M. (1996). Expressing functional domains of mouse calponin: Involvement of the region around alanine 145 in the actomyosin ATPase inhibitory activity of calponin. Biochemistry 35 (12), 3654–3661. 10.1021/bi952027e 8619984

[B18] Elsafadi M. Manikandan M. Almalki S. Mahmood A. Shinwari T. Vishnubalaji R. (2020). Transgelin is a poor prognostic factor associated with advanced colorectal cancer (CRC) stage promoting tumor growth and migration in a TGFβ-dependent manner. Cell Death Dis. 11 (5), 341. 10.1038/s41419-020-2529-6 32393769 PMC7214449

[B19] Elsafadi M. Manikandan M. Dawud R. A. Alajez N. M. Hamam R. Alfayez M. (2016). Transgelin is a TGFβ-inducible gene that regulates osteoblastic and adipogenic differentiation of human skeletal stem cells through actin cytoskeleston organization. Cell Death Dis. 7 (8), e2321. 10.1038/cddis.2016.196 27490926 PMC5108308

[B20] Facemire C. Brozovich F. V. Jin J. P. (2000). The maximal velocity of vascular smooth muscle shortening is independent of the expression of calponin. J. Muscle Res. Cell Motil. 21 (4), 367–373. 10.1023/a:1005680614296 11032347

[B21] Fattoum A. Roustan C. Smyczynski C. Der Terrossian E. Kassab R. (2003). Mapping the microtubule binding regions of calponin. Biochemistry 42 (5), 1274–1282. 10.1021/bi020336g 12564930

[B22] Feng H. Z. Wang H. Takahashi K. Jin J. P. (2019). Double deletion of calponin 1 and calponin 2 in mice decreases systemic blood pressure with blunted length-tension response of aortic smooth muscle. J. Mol. Cell Cardiol. 129, 49–57. 10.1016/j.yjmcc.2019.01.026 30707993 PMC6486848

[B23] Ferhat L. Esclapez M. Represa A. Fattoum A. Shirao T. Ben-Ari Y. (2003). Increased levels of acidic calponin during dendritic spine plasticity after pilocarpine-induced seizures. Hippocampus 13 (7), 845–858. 10.1002/hipo.10136 14620880

[B24] Ferjani I. Fattoum A. Maciver S. K. Bénistant C. Chahinian A. Manai M. (2006). A direct interaction with calponin inhibits the actin-nucleating activity of gelsolin. Biochem. J. 396 (3), 461–468. 10.1042/BJ20051690 16536729 PMC1482823

[B25] Flemming A. Huang Q. Q. Jin J. P. Jumaa H. Herzog S. (2015). A conditional knockout mouse model reveals that calponin-3 is dispensable for early B cell development. PLoS One 10 (6), e0128385. 10.1371/journal.pone.0128385 26046660 PMC4457629

[B26] Fraser E. D. Walsh M. P. (1995). Dephosphorylation of calponin by type 2B protein phosphatase. Biochemistry 34 (28), 9151–9158. 10.1021/bi00028a026 7619814

[B27] Fu Q. Liu P. C. Wang J. X. Song Q. S. Zhao X. F. (2009). Proteomic identification of differentially expressed and phosphorylated proteins in epidermis involved in larval-pupal metamorphosis of Helicoverpa armigera. BMC Genomics 10, 600. 10.1186/1471-2164-10-600 20003373 PMC2806347

[B28] Fu Y. Liu H. W. Forsythe S. M. Kogut P. McConville J. F. Halayko A. J. (2000). Mutagenesis analysis of human SM22: Characterization of actin binding. J. Appl. Physiol. 89(5), 1985–1990. 10.1152/jappl.2000.89.5.1985 11053353

[B29] Fujii T. Takagi H. Arimoto M. Ootani H. Ueeda T. (2000). Bundle formation of smooth muscle desmin intermediate filaments by calponin and its binding site on the desmin molecule. J. Biochem. 127 (3), 457–465. 10.1093/oxfordjournals.jbchem.a022628 10731718

[B30] Fujii T. Yabe S. Nakamura K. Koizumi Y. (2002). Functional analysis of rat acidic calponin. Biol. Pharm. Bull. 25 (5), 573–579. 10.1248/bpb.25.573 12033495

[B31] Galkin V. E. Orlova A. Fattoum A. Walsh M. P. Egelman E. H. (2006). The CH-domain of calponin does not determine the modes of calponin binding to F-actin. J. Mol. Biol. 359 (2), 478–485. 10.1016/j.jmb.2006.03.044 16626733

[B32] Gao J. Hwang J. M. Jin J. P. (1996). Complete nucleotide sequence, structural organization, and an alternatively spliced exon of mouse h1-calponin gene. Biochem. Biophys. Res. Commun. 218 (1), 292–297. 10.1006/bbrc.1996.0051 8573148

[B33] Gimona M. Djinovic-Carugo K. Kranewitter W. J. Winder S. J. (2002). Functional plasticity of CH domains. FEBS Lett. 513 (1), 98–106. 10.1016/s0014-5793(01)03240-9 11911887

[B34] Gimona M. Kaverina I. Resch G. P. Vignal E. Burgstaller G. (2003). Calponin repeats regulate actin filament stability and formation of podosomes in smooth muscle cells. Mol. Biol. Cell 14 (6), 2482–2491. 10.1091/mbc.e02-11-0743 12808045 PMC194896

[B35] Goetinck S. Waterston R. H. (1994). The *Caenorhabditis elegans* muscle-affecting gene unc-87 encodes a novel thin filament-associated protein. J. Cell Biol. 127 (1), 79–93. 10.1083/jcb.127.1.79 7929573 PMC2120179

[B36] Goodman A. Goode B. L. Matsudaira P. Fink G. R. (2003). The *Saccharomyces cerevisiae* calponin/transgelin homolog Scp1 functions with fimbrin to regulate stability and organization of the actin cytoskeleton. Mol. Biol. Cell 14 (7), 2617–2629. 10.1091/mbc.e03-01-0028 12857851 PMC165663

[B37] Gusev N. B. (2001). Some properties of caldesmon and calponin and the participation of these proteins in regulation of smooth muscle contraction and cytoskeleton formation. Biochem. (Mosc) 66 (10), 1112–1121. 10.1023/a:1012480829618 11736632

[B38] Haag J. Aigner T. (2007). Identification of calponin 3 as a novel Smad-binding modulator of BMP signaling expressed in cartilage. Exp. Cell Res. 313 (16), 3386–3394. 10.1016/j.yexcr.2007.08.003 17825283

[B39] Haeberle J. R. (1994). Calponin decreases the rate of cross-bridge cycling and increases maximum force production by smooth muscle myosin in an *in vitro* motility assay. J. Biol. Chem. 269 (17), 12424–12431. 10.1016/s0021-9258(18)99891-1 8175648

[B40] Han Y. H. Ryu K. B. Medina Jimenez B. I. Kim J. Lee H. Y. Cho S. J. (2020). Muscular development in urechis unicinctus (Echiura, Annelida). Int. J. Mol. Sci. 21 (7), 2306. 10.3390/ijms21072306 32225111 PMC7178014

[B41] Hines P. C. Gao X. White J. C. D'Agostino A. Jin J. P. (2014). A novel role of h2-calponin in regulating whole blood thrombosis and platelet adhesion during physiologic flow. Physiol. Rep. 2 (12), e12228. 10.14814/phy2.12228 25472609 PMC4332209

[B42] Hirata H. Ku W. C. Yip A. K. Ursekar C. P. Kawauchi K. Roy A. (2016). MEKK1-dependent phosphorylation of calponin-3 tunes cell contractility. J. Cell Sci. 129 (19), 3574–3582. 10.1242/jcs.189415 27528401

[B43] Hossain M. M. Crish J. F. Eckert R. L. Lin J. J. Jin J. P. (2005). h2-Calponin is regulated by mechanical tension and modifies the function of actin cytoskeleton. J. Biol. Chem. 280 (51), 42442–42453. 10.1074/jbc.M509952200 16236705 PMC1405912

[B44] Hossain M. M. Hwang D. Y. Huang Q. Q. Sasaki Y. Jin J. P. (2003). Developmentally regulated expression of calponin isoforms and the effect of h2-calponin on cell proliferation. Am. J. Physiol. Cell Physiol. 284 (1), C156–C167. 10.1152/ajpcell.00233.2002 12388067

[B45] Hossain M. M. Smith P. G. Wu K. Jin J. P. (2006). Cytoskeletal tension regulates both expression and degradation of h2-calponin in lung alveolar cells. Biochemistry 45 (51), 15670–15683. 10.1021/bi061718f 17176089 PMC1764619

[B46] Hossain M. M. Zhao G. Woo M. S. Wang J. H. Jin J. P. (2016). Deletion of calponin 2 in mouse fibroblasts increases myosin II-dependent cell traction force. Biochemistry 55 (43), 6046–6055. 10.1021/acs.biochem.6b00856 27733037

[B47] Hsieh T. B. Feng H. Z. Jin J. P. (2021). Deletion of calponin 2 reduces the formation of postoperative peritoneal adhesions. J. Invest. Surg. 35, 517–524. 10.1080/08941939.2021.1880672 33622156 PMC8751165

[B48] Hsieh T. B. Jin J. P. (2022). Loss of Calponin 2 causes age-progressive proteinuria in mice. Physiol. Rep. 10 (18), e15370. 10.14814/phy2.15370 36117313 PMC9483440

[B49] Huang Q. Q. Hossain M. M. Sun W. Xing L. Pope R. M. Jin J. P. (2016). Deletion of calponin 2 in macrophages attenuates the severity of inflammatory arthritis in mice. Am. J. Physiol. Cell Physiol. 311 (4), C673–C685. 10.1152/ajpcell.00331.2015 27488671 PMC5129749

[B50] Huang Q. Q. Hossain M. M. Wu K. Parai K. Pope R. M. Jin J. P. (2008). Role of H2-calponin in regulating macrophage motility and phagocytosis. J. Biol. Chem. 283 (38), 25887–25899. 10.1074/jbc.M801163200 18617524 PMC2533796

[B51] Ichikawa K. Ito M. Okubo S. Konishi T. Nakano T. Mino T. (1993). Calponin phosphatase from smooth muscle: A possible role of type 1 protein phosphatase in smooth muscle relaxation. Biochem. Biophys. Res. Commun. 193 (3), 827–833. 10.1006/bbrc.1993.1700 8391807

[B52] Jang S. H. Jun C. D. Park Z. Y. (2015). Label-free quantitative phosphorylation analysis of human transgelin2 in Jurkat T cells reveals distinct phosphorylation patterns under PKA and PKC activation conditions. Proteome Sci. 13, 14. 10.1186/s12953-015-0070-9 25844069 PMC4384351

[B53] Jiang W. R. Cady G. Hossain M. M. Huang Q. Q. Wang X. Jin J. P. (2014). Mechanoregulation of h2-calponin gene expression and the role of Notch signaling. J. Biol. Chem. 289 (3), 1617–1628. 10.1074/jbc.M113.498147 24285540 PMC3894341

[B54] Jin J. P. Walsh M. P. Resek M. E. McMartin G. A. (1996). Expression and epitopic conservation of calponin in different smooth muscles and during development. Biochem. Cell Biol. 74 (2), 187–196. 10.1139/o96-019 9213427

[B55] Jin J. P. Walsh M. P. Sutherland C. Chen W. (2000). A role for serine-175 in modulating the molecular conformation of calponin. Biochem. J. 350, 579–588. 10.1042/bj3500579 10947974 PMC1221287

[B56] Jin J. P. Wu D. Gao J. Nigam R. Kwong S. (2003). Expression and purification of the h1 and h2 isoforms of calponin. Protein Expr. Purif. 31 (2), 231–239. 10.1016/s1046-5928(03)00185-2 14550641

[B57] Jin J. P. Zhang Z. Bautista J. A. (2008). Isoform diversity, regulation, and functional adaptation of troponin and calponin. Crit. Rev. Eukaryot. Gene Expr. 18 (2), 93–124. 10.1615/critreveukargeneexpr.v18.i2.10 18304026

[B58] Jo S. Kim H. R. Mun Y. Jun C. D. (2018). Transgelin-2 in immunity: Its implication in cell therapy. J. Leukoc. Biol. 104 (5), 903–910. 10.1002/JLB.MR1117-470R 29749649

[B59] Jones M. K. Yang W. McManus D. P. (2001). Immunolocalization of the 38.3 kDa calponin-like protein in stratified muscles of the tail of Schistosoma japonicum cercariae. Parasitol. Int. 50 (2), 129–133. 10.1016/s1383-5769(01)00060-5 11438435

[B60] Junghans D. Herzog S. (2018). Cnn3 regulates neural tube morphogenesis and neuronal stem cell properties. FEBS J. 285 (2), 325–338. 10.1111/febs.14338 29151265

[B61] Kato R. Hayashi M. Aiuchi T. Sawada N. Obama T. Itabe H. (2019). Temporal and spatial changes of peroxiredoxin 2 levels in aortic media at very early stages of atherosclerotic lesion formation in apoE-knockout mice. Free Radic. Biol. Med. 130, 348–360. 10.1016/j.freeradbiomed.2018.10.458 30395970

[B62] Kawasaki H. Kretsinger R. H. (2017). Structural and functional diversity of EF-hand proteins: Evolutionary perspectives. Protein Sci. 26 (10), 1898–1920. 10.1002/pro.3233 28707401 PMC5606533

[B63] Kim H. R. Gallant C. Morgan K. G. (2013). Regulation of PKC autophosphorylation by calponin in contractile vascular smooth muscle tissue. Biomed. Res. Int. 2013, 358643. 10.1155/2013/358643 24350264 PMC3852320

[B64] Korenbaum E. Rivero F. (2002). Calponin homology domains at a glance. J. Cell Sci. 115 (18), 3543–3545. 10.1242/jcs.00003 12186940

[B65] Kuo H. C. Chiu C. C. Chang W. C. Sheen J. M. Ou C. Y. (2011). Use of proteomic differential displays to assess functional discrepancies and adjustments of human bone marrow- and Wharton jelly-derived mesenchymal stem cells. J. Proteome Res. 10 (3), 1305–1315. 10.1021/pr101057w 21155588

[B66] Lawson D. Harrison M. Shapland C. (1997). Fibroblast transgelin and smooth muscle SM22alpha are the same protein, the expression of which is down-regulated in many cell lines. Cell Motil. Cytoskelet. 38 (3), 250–257. 10.1002/(SICI)1097-0169(1997)38:3<250::AID-CM3>3.0.CO;2-9 9384215

[B67] Lees-Miller J. P. Heeley D. H. Smillie L. B. Kay C. M. (1987). Isolation and characterization of an abundant and novel 22-kDa protein (SM22) from chicken gizzard smooth muscle. J. Biol. Chem. 262 (7), 2988–2993. 10.1016/s0021-9258(18)61457-7 3818630

[B68] Leinweber B. Parissenti A. M. Gallant C. Gangopadhyay S. S. Kirwan-Rhude A. Leavis P. C. (2000). Regulation of protein kinase C by the cytoskeletal protein calponin. J. Biol. Chem. 275 (51), 40329–40336. 10.1074/jbc.M008257200 11006297

[B69] Leinweber B. D. Leavis P. C. Grabarek Z. Wang C. L. Morgan K. G. (1999). Extracellular regulated kinase (ERK) interaction with actin and the calponin homology (CH) domain of actin-binding proteins. Biochem. J. 344, 117–123. 10.1042/bj3440117 10548541 PMC1220621

[B70] Lew Z. X. Zhou H. M. Fang Y. Y. Ye Z. Zhong W. Yang X. Y. (2020). Transgelin interacts with PARP1 in human colon cancer cells. Cancer Cell Int. 20, 366. 10.1186/s12935-020-01461-y 32774160 PMC7398379

[B71] Li M. Li S. Lou Z. Liao X. Zhao X. Meng Z. (2008). Crystal structure of human transgelin. J. Struct. Biol. 162 (2), 229–236. 10.1016/j.jsb.2008.01.005 18291675

[B72] Lin Y. Buckhaults P. J. Lee J. R. Xiong H. Farrell C. Podolsky R. H. (2009). Association of the actin-binding protein transgelin with lymph node metastasis in human colorectal cancer. Neoplasia 11 (9), 864–873. 10.1593/neo.09542 19724680 PMC2735797

[B73] Liu J. Zhang Y. Li Q. Wang Y. (2020). Transgelins: Cytoskeletal associated proteins implicated in the metastasis of colorectal cancer. Front. Cell Dev. Biol. 8, 573859. 10.3389/fcell.2020.573859 33117801 PMC7575706

[B74] Liu R. Hossain M. M. Chen X. Jin J. P. (2017). Mechanoregulation of sm22α/transgelin. Biochemistry 56 (41), 5526–5538. 10.1021/acs.biochem.7b00794 28898058

[B75] Liu R. Jin J. P. (2016). Calponin isoforms CNN1, CNN2 and CNN3: Regulators for actin cytoskeleton functions in smooth muscle and non-muscle cells. Gene 585 (1), 143–153. 10.1016/j.gene.2016.02.040 26970176 PMC5325697

[B76] Liu R. Jin J. P. (2016). Deletion of calponin 2 in macrophages alters cytoskeleton-based functions and attenuates the development of atherosclerosis. J. Mol. Cell Cardiol. 99, 87–99. 10.1016/j.yjmcc.2016.08.019 27575021 PMC5325694

[B77] Ma Y. Bogatcheva N. V. Gusev N. B. (2000). Heat shock protein (hsp90) interacts with smooth muscle calponin and affects calponin-binding to actin. Biochim. Biophys. Acta 1476 (2), 300–310. 10.1016/s0167-4838(99)00250-2 10669794

[B78] Maselli V. Galdiero E. Salzano A. M. Scaloni A. Maione A. Falanga A. (2020). OctoPartenopin: Identification and preliminary characterization of a novel antimicrobial peptide from the suckers of *Octopus vulgaris* . Mar. Drugs 18 (8), 380. 10.3390/md18080380 32717885 PMC7460285

[B79] Masuda H. Tanaka K. Takagi M. Ohgami K. Sakamaki T. Shibata N. (1996). Molecular cloning and characterization of human non-smooth muscle calponin. J. Biochem. 120 (2), 415–424. 10.1093/oxfordjournals.jbchem.a021428 8889829

[B80] Matsui T. S. Ishikawa A. Deguchi S. (2018). Transgelin-1 (SM22α) interacts with actin stress fibers and podosomes in smooth muscle cells without using its actin binding site. Biochem. Biophys. Res. Commun. 505 (3), 879–884. 10.1016/j.bbrc.2018.09.176 30301526

[B144] Matthew J. D. Khromov A. S. McDuffie M. J. Somlyo A. V. Somlyo A. P. Taniguchi S. (2000). Contractile properties and proteins of smooth muscles of a calponin knockout mouse. J Physiol. 529 (3), 811–24. 10.1111/j.1469-7793.2000.00811.x 11118508 PMC2270213

[B81] Meng T. Liu L. Hao R. Chen S. Dong Y. (2017). Transgelin-2: A potential oncogenic factor. Tumour Biol. 39 (6), 1010428317702650. 10.1177/1010428317702650 28639888

[B82] Menice C. B. Hulvershorn J. Adam L. P. Wang C. A. Morgan K. G. (1997). Calponin and mitogen-activated protein kinase signaling in differentiated vascular smooth muscle. J. Biol. Chem. 272 (40), 25157–25161. 10.1074/jbc.272.40.25157 9312127

[B83] Meyer-Rochow V. B. Fraile B. Paniagua R. Royuela M. (2003). First immunocytochemical study of echinoderm smooth muscle: The antarctic cushionstar odontaster validus koehler (echinodermata, asteroidea). Protoplasma 220 (3-4), 227–232. 10.1007/s00709-002-0048-1 12664287

[B84] Mezgueldi M. Fattoum A. Derancourt J. Kassab R. (1992). Mapping of the functional domains in the amino-terminal region of calponin. J. Biol. Chem. 267 (22), 15943–15951. 10.1016/s0021-9258(19)49625-7 1639822

[B85] Moazzem Hossain M. Wang X. Bergan R. C. Jin J. P. (2014). Diminished expression of h2-calponin in prostate cancer cells promotes cell proliferation, migration and the dependence of cell adhesion on substrate stiffness. FEBS Open Bio 4, 627–636. 10.1016/j.fob.2014.06.003 PMC4141211 25161871

[B86] Moralez I. Phelps A. Riley B. Raines M. Wirrig E. Snarr B. (2006). Muscularizing tissues in the endocardial cushions of the avian heart are characterized by the expression of h1-calponin. Dev. Dyn. 235 (6), 1648–1658. 10.1002/dvdy.20738 16502418

[B87] Na B. R. Kim H. R. Piragyte I. Oh H. M. Kwon M. S. Akber U. (2015). TAGLN2 regulates T cell activation by stabilizing the actin cytoskeleton at the immunological synapse. J. Cell Biol. 209 (1), 143–162. 10.1083/jcb.201407130 25869671 PMC4395477

[B88] Nakano K. Bunai F. Numata O. (2005). Stg 1 is a novel SM22/transgelin-like actin-modulating protein in fission yeast. FEBS Lett. 579 (28), 6311–6316. 10.1016/j.febslet.2005.10.011 16256112

[B89] Nigam R. Triggle C. R. Jin J. P. (1998). h1-and h2-calponins are not essential for norepinephrine- or sodium fluoride-induced contraction of rat aortic smooth muscle. J. Muscle Res. Cell Motil. 19 (6), 695–703. 10.1023/a:1005389300151 9742453

[B90] Ono K. Obinata T. Yamashiro S. Liu Z. Ono S. (2015). UNC-87 isoforms, *Caenorhabditis elegans* calponin-related proteins, interact with both actin and myosin and regulate actomyosin contractility. Mol. Biol. Cell 26 (9), 1687–1698. 10.1091/mbc.E14-10-1483 25717181 PMC4436780

[B91] Ono S. (2021). Diversification of the calponin family proteins by gene amplification and repeat expansion of calponin-like motifs. Cytoskelet. Hob. 78 (5), 199–205. 10.1002/cm.21683 PMC8958760 34333878

[B92] Oonuma K. Yamamoto M. Moritsugu N. Okawa N. Mukai M. Sotani M. (2021). Evolution of developmental programs for the midline structures in chordates: Insights from gene regulation in the floor plate and hypochord homologues of Ciona embryos. Front. Cell Dev. Biol. 9, 704367. 10.3389/fcell.2021.704367 34235159 PMC8256262

[B93] Parker C. A. Takahashi K. Tao T. Morgan K. G. (1994). Agonist-induced redistribution of calponin in contractile vascular smooth muscle cells. Am. J. Physiol. 267 (5), C1262–C1270. 10.1152/ajpcell.1994.267.5.C1262 7526695

[B94] Pearlstone J. R. Weber M. Lees-Miller J. P. Carpenter M. R. Smillie L. B. (1987). Amino acid sequence of chicken gizzard smooth muscle SM22 alpha. J. Biol. Chem. 262 (13), 5985–5991. 10.1016/s0021-9258(18)45526-3 3571244

[B95] Plazyo O. Hao W. Jin J. P. (2020). The absence of calponin 2 in rabbits suggests caution in choosing animal models. Front. Bioeng. Biotechnol. 8, 42. 10.3389/fbioe.2020.00042 32185166 PMC7058930

[B96] Plazyo O. Liu R. Moazzem Hossain M. Jin J. P. (2018). Deletion of calponin 2 attenuates the development of calcific aortic valve disease in ApoE(-/-) mice. J. Mol. Cell Cardiol. 121, 233–241. 10.1016/j.yjmcc.2018.07.249 30053524

[B97] Plazyo O. Sheng J. J. Jin J. P. (2019). Downregulation of calponin 2 contributes to the quiescence of lung macrophages. Am. J. Physiol. Cell Physiol. 317 (4), C749–C761. 10.1152/ajpcell.00036.2019 31365293 PMC6850996

[B98] Plesch B. (1977). An ultrastructural study of the musculature of the pond snail *Lymnaea stagnalis* (l). Cell Tissue Res. 180 (3), 317–340. 10.1007/BF00227599 872199

[B99] Qian A. Hsieh T. B. Hossain M. M. Lin J. J. Jin J. P. (2021). A rapid degradation of calponin 2 is required for cytokinesis. Am. J. Physiol. Cell Physiol. 321 (2), C355–C368. 10.1152/ajpcell.00569.2020 34133238 PMC8526346

[B100] Qiu Z. Chu Y. Xu B. Wang Q. Jiang M. Li X. (2017). Increased expression of calponin 2 is a positive prognostic factor in pancreatic ductal adenocarcinoma. Oncotarget 8 (34), 56428–56442. 10.18632/oncotarget.17701 28915602 PMC5593573

[B101] Ren W. Z. Ng G. Y. Wang R. X. Wu P. H. O'Dowd B. F. Osmond D. H. (1994). The identification of NP25: A novel protein that is differentially expressed by neuronal subpopulations. Brain Res. Mol. Brain Res. 22 (1-4), 173–185. 10.1016/0169-328x(94)90045-0 8015377

[B102] Royuela M. Fraile B. Arenas M. I. Paniagua R. (2000). Characterization of several invertebrate muscle cell types: A comparison with vertebrate muscles. Microsc. Res. Tech. 48 (2), 107–115. 10.1002/(SICI)1097-0029(20000115)48:2<107::AID-JEMT6>3.0.CO;2-U 10649511

[B103] Royuela M. Fraile B. Picazo M. L. Paniagua R. (1997). Immunocytochemical electron microscopic study and Western blot analysis of caldesmon and calponin in striated muscle of the fruit fly *Drosophila melanogaster* and in several muscle cell types of the earthworm Eisenia foetida. Eur. J. Cell Biol. 72 (1), 90–94. 9013730

[B104] Samaha F. F. Ip H. S. Morrisey E. E. Seltzer J. Tang Z. Solway J. (1996). Developmental pattern of expression and genomic organization of the calponin-h1 gene. A contractile smooth muscle cell marker. J. Biol. Chem. 271 (1), 395–403. 10.1074/jbc.271.1.395 8550594

[B105] Sayar N. Karahan G. Konu O. Bozkurt B. Bozdogan O. Yulug I. G. (2015). Transgelin gene is frequently downregulated by promoter DNA hypermethylation in breast cancer. Clin. Epigenetics 7, 104. 10.1186/s13148-015-0138-5 26421063 PMC4587865

[B106] Sharma A. Dagar S. Mylavarapu S. V. S. (2020). Transgelin-2 and phosphoregulation of the LIC2 subunit of dynein govern mitotic spindle orientation. J. Cell Sci. 133 (12), jcs239673. 10.1242/jcs.239673 32467330

[B107] She Y. Li C. Jiang T. Lei S. Zhou S. Shi H. (2021). Knockdown of CNN3 impairs myoblast proliferation, differentiation, and protein synthesis via the mTOR pathway. Front. Physiol. 12, 659272. 10.3389/fphys.2021.659272 34305633 PMC8295729

[B108] Sheehan G. Kavanagh K. (2018). Analysis of the early cellular and humoral responses of Galleria mellonella larvae to infection by Candida albicans. Virulence 9 (1), 163–172. 10.1080/21505594.2017.1370174 28872999 PMC5955201

[B109] Shi J. Ren M. She X. Zhang Z. Zhao Y. Han Y. (2020). Transgelin-2 contributes to proliferation and progression of hepatocellular carcinoma via regulating Annexin A2. Biochem. Biophys. Res. Commun. 523 (3), 632–638. 10.1016/j.bbrc.2020.01.028 31941608

[B110] Shibukawa Y. Yamazaki N. Daimon E. Wada Y. (2013). Rock-dependent calponin 3 phosphorylation regulates myoblast fusion. Exp. Cell Res. 319 (5), 633–648. 10.1016/j.yexcr.2012.12.022 23276748

[B111] Shibukawa Y. Yamazaki N. Kumasawa K. Daimon E. Tajiri M. Okada Y. (2010). Calponin 3 regulates actin cytoskeleton rearrangement in trophoblastic cell fusion. Mol. Biol. Cell 21 (22), 3973–3984. 10.1091/mbc.E10-03-0261 20861310 PMC2982094

[B112] Shishibori T. Yamashita K. Bandoh J. Oyama Y. Kobayashi R. (1996). Presence of Ca(2+)-sensitive and -insensitive SM22 alpha isoproteins in bovine aorta. Biochem. Biophys. Res. Commun. 229 (1), 225–230. 10.1006/bbrc.1996.1784 8954110

[B113] Sirenko V. V. Dobrzhanskaya A. V. Shelud'ko N. S. Borovikov Y. S. (2016). Calponin-like protein from mussel smooth muscle is a competitive inhibitor of actomyosin ATPase. Biochem. (Mosc) 81 (1), 28–33. 10.1134/S000629791601003X 26885580

[B114] Sirenko V. V. Simonyan A. H. Dobrzhanskaya A. V. Shelud'ko N. S. Borovikov Y. S. (2013). 40-kDa protein from thin filaments of the mussel Crenomytilus grayanus changes the conformation of F-actin during the ATPase cycle. Biochem. (Mosc) 78 (3), 273–281. 10.1134/S0006297913030097 23586721

[B115] Springer M. L. Ozawa C. R. Blau H. M. (2002). Transient production of alpha-smooth muscle actin by skeletal myoblasts during differentiation in culture and following intramuscular implantation. Cell Motil. Cytoskelet. 51 (4), 177–186. 10.1002/cm.10022 11977092

[B116] Stork N. E. (2018). How many species of insects and other terrestrial arthropods are there on earth? Annu. Rev. Entomol. 63, 31–45. 10.1146/annurev-ento-020117-043348 28938083

[B117] Stradal T. Kranewitter W. Winder S. J. Gimona M. (1998). CH domains revisited. FEBS Lett. 431 (2), 134–137. 10.1016/s0014-5793(98)00751-0 9708889

[B118] Strasser P. Gimona M. Moessler H. Herzog M. Small J. V. (1993). Mammalian calponin. Identification and expression of genetic variants. FEBS Lett. 330 (1), 13–18. 10.1016/0014-5793(93)80909-e 8370452

[B119] Su N. Chen M. Chen S. Li C. Xie Y. Zhu Y. (2013). Overexpression of H1 calponin in osteoblast lineage cells leads to a decrease in bone mass by disrupting osteoblast function and promoting osteoclast formation. J. Bone Min. Res. 28 (3), 660–671. 10.1002/jbmr.1778 PMC3716280 23044709

[B120] Takahashi K. Abe M. Hiwada K. Kokubu T. (1988). A novel troponin T-like protein (calponin) in vascular smooth muscle: Interaction with tropomyosin paracrystals. J. Hypertens. Suppl. 6 (4), S40–S43. 10.1097/00004872-198812040-00008 3241227

[B121] Takahashi K. Hiwada K. Kokubu T. (1986). Isolation and characterization of a 34,000-dalton calmodulin- and F-actin-binding protein from chicken gizzard smooth muscle. Biochem. Biophys. Res. Commun. 141 (1), 20–26. 10.1016/s0006-291x(86)80328-x 3606745

[B122] Takahashi K. Yoshimoto R. Fuchibe K. Fujishige A. Mitsui-Saito M. Hori M. (2000). Regulation of shortening velocity by calponin in intact contracting smooth muscles. Biochem. Biophys. Res. Commun. 279 (1), 150–157. 10.1006/bbrc.2000.3909 11112431

[B123] Tang D. C. Kang H. M. Jin J. P. Fraser E. D. Walsh M. P. (1996). Structure-function relations of smooth muscle calponin. The critical role of serine 175. J. Biol. Chem. 271 (15), 8605–8611. 10.1074/jbc.271.15.8605 8621490

[B124] Tang J. Hu G. Hanai J. Yadlapalli G. Lin Y. Zhang B. (2006). A critical role for calponin 2 in vascular development. J. Biol. Chem. 281 (10), 6664–6672. 10.1074/jbc.M506991200 16317011

[B125] Thompson O. Moghraby J. S. Ayscough K. R. Winder S. J. (2012). Depletion of the actin bundling protein SM22/transgelin increases actin dynamics and enhances the tumourigenic phenotypes of cells. BMC Cell Biol. 13, 1. 10.1186/1471-2121-13-1 22257561 PMC3280177

[B126] Ulmer B. Hagenlocher C. Schmalholz S. Kurz S. Schweickert A. Kohl A. (2013). Calponin 2 acts as an effector of noncanonical Wnt-mediated cell polarization during neural crest cell migration. Cell Rep. 3 (3), 615–621. 10.1016/j.celrep.2013.02.015 23499442

[B127] Verone A. R. Duncan K. Godoy A. Yadav N. Bakin A. Koochekpour S. (2013). Androgen-responsive serum response factor target genes regulate prostate cancer cell migration. Carcinogenesis 34 (8), 1737–1746. 10.1093/carcin/bgt126 23576568 PMC3731805

[B128] Wage J. Lerebours A. Hardege J. D. Rotchell J. M. (2016). Exposure to low pH induces molecular level changes in the marine worm, Platynereis dumerilii. Ecotoxicol. Environ. Saf. 124, 105–110. 10.1016/j.ecoenv.2015.10.008 26476878

[B129] Walsh M. P. (1991). The Ayerst Award Lecture 1990. Calcium-dependent mechanisms of regulation of smooth muscle contraction. Biochem. Cell Biol. 69 (12), 771–800. 10.1139/o91-119 1818584

[B130] Winder S. J. Jess T. Ayscough K. R. (2003). SCP1 encodes an actin-bundling protein in yeast. Biochem. J. 375 (2), 287–295. 10.1042/BJ20030796 12868959 PMC1223692

[B131] Wu J. Wang Y. Y. Yang X. W. Zhang X. T. Tang J. Y. (2021). Biochemical features and physiological roles of hNP22 in the central nervous system. Front. Cell Dev. Biol. 9, 634710. 10.3389/fcell.2021.634710 33748120 PMC7969789

[B132] Wu K. C. Jin J. P. (2008). Calponin in non-muscle cells. Cell Biochem. Biophys. 52 (3), 139–148. 10.1007/s12013-008-9031-6 18946636

[B133] Yamane T. Asanoma K. Kobayashi H. Liu G. Yagi H. Ohgami T. (2015). Identification of the critical site of calponin 1 for suppression of ovarian cancer properties. Anticancer Res. 35 (11), 5993–5999. 26504022

[B134] Yamashiro S. Gimona M. Ono S. (2007). UNC-87, a calponin-related protein in *C. elegans*, antagonizes ADF/cofilin-mediated actin filament dynamics. J. Cell Sci. 120 (17), 3022–3033. 10.1242/jcs.013516 17684058 PMC2365702

[B135] Yang Z. Chang Y. J. Miyamoto H. Ni J. Niu Y. Chen Z. (2007). Transgelin functions as a suppressor via inhibition of ARA54-enhanced androgen receptor transactivation and prostate cancer cell growth. Mol. Endocrinol. 21 (2), 343–358. 10.1210/me.2006-0104 17082327

[B136] Yin L. M. Ulloa L. Yang Y. Q. (2019). Transgelin-2: Biochemical and clinical implications in cancer and asthma. Trends Biochem. Sci. 44 (10), 885–896. 10.1016/j.tibs.2019.05.004 31256982 PMC7023894

[B137] Yin L. M. Xu Y. D. Peng L. L. Duan T. T. Liu J. Y. Xu Z. (2018). Transgelin-2 as a therapeutic target for asthmatic pulmonary resistance. Sci. Transl. Med. 10 (427), eaam8604. 10.1126/scitranslmed.aam8604 29437149 PMC6310021

[B138] Zhang H. Jiang M. Liu Q. Han Z. Zhao Y. Ji S. (2018). miR-145-5p inhibits the proliferation and migration of bladder cancer cells by targeting TAGLN2. Oncol. Lett. 16 (5), 6355–6360. 10.3892/ol.2018.9436 30405771 PMC6202496

[B139] Zhang X. Meng H. Wang M. M. (2013). Collagen represses canonical Notch signaling and binds to Notch ectodomain. Int. J. Biochem. Cell Biol. 45 (7), 1274–1280. 10.1016/j.biocel.2013.03.020 23579095 PMC3683383

[B140] Zhang Y. Ye Y. Shen D. Jiang K. Zhang H. Sun W. (2010). Identification of transgelin-2 as a biomarker of colorectal cancer by laser capture microdissection and quantitative proteome analysis. Cancer Sci. 101 (2), 523–529. 10.1111/j.1349-7006.2009.01424.x 19930159 PMC11159707

[B141] Zhong L. He X. Si X. Wang H. Li B. Hu Y. (2019). SM22α (smooth muscle 22α) prevents aortic aneurysm formation by inhibiting smooth muscle cell phenotypic switching through suppressing reactive oxygen species/NF-κB (nuclear factor-κb). Arterioscler. Thromb. Vasc. Biol. 39 (1), e10–e25. 10.1161/ATVBAHA.118.311917 30580562

[B142] Zhou H. Di Palma S. Preisinger C. Peng M. Polat A. N. Heck A. J. R. (2013). Toward a comprehensive characterization of a human cancer cell phosphoproteome. J. Proteome Res. 12 (1), 260–271. 10.1021/pr300630k 23186163

[B143] Zhu Q. Emanuele N. V. Van Thiel D. H. (2004). Calponin is expressed by Sertoli cells within rat testes and is associated with actin-enriched cytoskeleton. Cell Tissue Res. 316 (2), 243–253. 10.1007/s00441-004-0864-z 14997380

